# Objective assessment of eye alignment and disparity-driven vergence in Parkinson’s disease

**DOI:** 10.3389/fnagi.2023.1217765

**Published:** 2023-10-31

**Authors:** Palak Gupta, Jordan M. Murray, Sinem Balta Beylergil, Jonathan Jacobs, Camilla W. Kilbane, Aasef G. Shaikh, Fatema F. Ghasia

**Affiliations:** ^1^Department of Biomedical Engineering, Case Western Reserve University, Cleveland, OH, United States; ^2^Visual Neurosciences and Ocular Motility Laboratory, Cole Eye Institute, Cleveland Clinic, Cleveland, OH, United States; ^3^Daroff-Dell’Osso Ocular Motility Laboratory, Cleveland VA Medical Center, Cleveland, OH, United States; ^4^Department of Neurology, University Hospitals, Cleveland, OH, United States; ^5^Neurology Service, Louis Stokes Cleveland VA Medical Center, Cleveland, OH, United States

**Keywords:** Parkinson’s disease, basal ganglia, supra-oculomotor area, strabismus, vergence, density-based clustering

## Abstract

**Background:**

Self-reported diplopia is described in up to one-third of Parkinson’s disease (PD) patients.

**Objective:**

The purpose of our study was to expand our understanding of the mechanistic underpinnings of diplopia in PD. We hypothesize that the time-based control of eye alignment and increased eye deviation under binocular viewing will be related to the fusion-initiating and fusion-maintaining component deficits of disparity-driven vergence in PD.

**Methods:**

We used high-resolution video-oculography to measure eye alignment under binocular and monocular viewing and disparity-driven vergence in 33 PD and 10 age-matched healthy participants. We computed eye deviation and time-based control of eye alignment, occurrence of conjugate saccadic eye movements, latency and gain of vergence (fusion initiation), and variance of eye position at the end of dynamic vergence (fusion maintenance).

**Results:**

We categorized PD subjects into three groups, considering their time-based control of eye alignment as compared to healthy controls in binocular viewing. Group 1 = 45% had good control and spent >80% of the time when the eyes were well-aligned, Group 2 = 26% had intermediate control and spent <80% but greater >5% of the time when the eyes were well-aligned, and Group 3 = 29% had very poor control with increased eye deviation majority of the times (<5% of the time when the eyes were well-aligned). All three groups exhibited greater eye deviation under monocular viewing than controls. PD subjects exhibited fusion-initiating and fusion-maintaining vergence deficits (prolonged latencies, reduced vergence gain, increased variance of fusion-maintaining component) with a greater probability of saccadic movements than controls. Group 2 and Group 3 subjects were more likely to exhibit failure to initiate vergence (>20%) than Group 1 (13%) and controls (0%) trials. No significant difference was found in the Unified Parkinson’s Disease Rating Scale (UPDRS—a tool to measure the severity of PD) values between the three PD groups (Group 1 = 33.69 ± 14.22, Group 2 = 38.43 ± 22.61, and Group 3 = 23.44 ± 1, *p* > 0.05).

**Conclusion:**

The majority of PD subjects within our cohort had binocular dysfunction with increased eye deviation under monocular viewing and disparity-driven vergence deficits. PD subjects with intermediate or poor control of eye deviation under binocular viewing had greater fusion-initiating and fusion-maintaining vergence deficits. The study highlights the importance of assessing binocular dysfunction in PD subjects independent of the severity of motor symptoms.

## Introduction

Parkinson’s disease (PD) is a neurodegenerative condition characterized by a host of motor and non-motor symptoms affecting about 10 million individuals worldwide. Visual impairments in PD are far more common than appreciated and include blepharospasm, dry eyes, reduced blinking, visual hallucinations, decreased visual acuity, and contrast sensitivity (Repka et al., 1996; Biousse et al., 2004; Almer et al., 2012; Anderson and MacAskill, 2013; Borm et al., 2020). On the oculomotor level, the deficits comprise abnormal rapid gaze shifts (i.e., saccades) with increased saccadic intrusions causing difficulties in reading and scanning the visual surroundings (Lepore, 2006; Almer et al., 2012; Urwyler et al., 2014; Antoniades et al., 2015; Armstrong, 2015; Beylergil et al., 2022). Up to 70% of PD patients experience some form of visual impairment as per the self-reported visual function and non-motor symptom questionnaires (Schindlbeck et al., 2017; Visser et al., 2019; Borm et al., 2020; Smilowska et al., 2020; Hamedani et al., 2021; Naumann et al., 2021). Vergence insufficiency, the disconjugate movement of the eyes in response to a target jump from far to near and vice versa, has been reported in PD (Racette et al., 1999; Biousse et al., 2004; Lepore, 2006; Urwyler et al., 2014; Armstrong, 2015). Ocular misalignment is described as both a consequence of convergence insufficiency (Repka et al., 1996; Kang et al., 2018) as well as a more nuanced subcortical deficit in Parkinson’s disease (Almer et al., 2012; Searle and Rowe, 2016; Naumann et al., 2021).

There are two key sensory drives for inducing vergence while looking at targets at different depths, which are (1) visual blur under monocular viewing that induces accommodative vergence; and (2) retinal disparity under binocular viewing, i.e., differences in the spatial position of the image on two retinas that induces disparity-driven vergence. There are two key motor components of disparity-driven vergence, which are (1) the fusion initiation component, i.e., the open loop or pulse of neural activity facilitating the initial movement responsible for vergence, and (2) the fusion maintenance component driven by visual or internal feedback to align the eyes accurately to maintain a fusion of the binocular image of the target (Semmlow et al., 2019, 2021). A handful of studies have quantified vergence in PD and have reported increased latency with variable effects on convergence velocity and gain (Hanuška et al., 2015; Gupta et al., 2021a,b). None of the studies to date have examined eye alignment and disparity-driven vergence using eye movement recordings in the same cohort. The overarching goal of this study is to examine the relationship between the impairments in fusion-initiating and fusion-maintaining components of vergence and eye misalignment, i.e., strabismus in PD. We hypothesize that the time-based control of eye alignment and increased eye deviation under binocular viewing will be related to the fusion-initiating and fusion-maintaining component deficits of disparity-driven vergence in PD.

## Methods

### Study participants and experiment protocol

The institutional review board of the Cleveland Clinic approved the protocol, and written informed consent was obtained from each participant in accordance with the Declaration of Helsinki. We recruited 33 PD patients (age: 69.16 ± 8.2 years) who were referred by neurologists and 10 healthy controls (age: 65.14 ± 6.8 years). PD diagnosis was based on clinical impression and the UK Brain Bank criteria. Of the PD subjects recruited in our study, only 15% of recruited subjects had self-reported diplopia. Table 1 summarizes the demographical information and clinical neurological parameters of recruited subjects. The visual acuity and stereo-acuity, refraction, and strabismus angle measurements at distance and near the time of eye movement recordings were noted. The presence of strabismus was assessed with a prism and alternate cover test in the appropriate diagnostic fields of gaze at 6 m as well as at one-third meter in the primary position. We used the standard guidelines recommended by the pediatric ophthalmology and strabismus subsection of the American Academy of Ophthalmology Preferred Practice Pattern to assess strabismus. Near point of convergence (NPC) was measured with a Prince rule. Out of the 33 PD patients, only 2 patients did not have clinically measurable exodeviation at near. Only 1 control subject had a small intermittent exodeviation of 2 prism diopters at near, whereas the remaining age-matched controls did not have any clinically measured exodeviation at near. We defined normal intact vergence as a near point convergence (NPC) of less than 10 cm. Out of the 6 healthy age-matched controls, all had intact vergence whereas one control had mild increase in NPC at 14 cm. In our study, all PD patients exhibited increased near point of convergence (NPC > 10 cm).

**Table 1:** Clinical and demographical details of PD patients studied.

Subject group	Subject ID	Age	Sex	Disease duration (y)	Major complaint	Falls over last 6 months^a^	UPDRS
1	PD01	76	M	11	Tremor	7	51
1	PD03	52	M	12	Arm tremor	0	40.5
1	PD09	79	M	17	Hand tremor	2	54
1	PD10	69	M	8	Pain on the left side	0	27
1	PD11	64	M	10	Left hand tremor	>20	23.5
1	PD12	62	M	10	Memory, fatigue, tremor	4	30
1	PD13	68	M	15	Non-motor (Swallowing, speech)	0	50.5
1	PD19	75	M	10+	Dyskinesia, tremor		32.5
1	PD21	65	F	12	Tremor	0	49.5
1	PD22	69	M	5	Tremor	0	25
1	PD27	75	M	10	Fatigue	3	10.5
1	PD29	70	F	9	Limb tremor	0	15.5
1	PD31	74	M	4	Tremor	0	
1	PD32	78	M	6	Head tremor	0	28.5
1	PD33	72	M	4	Hand tremor	0	39
2	PD05	69	M	9	Tremor, leg weakness, sleep problems	4	48.5
2	PD07	64	M	18	Speech, handwriting	0	76
2	PD08	71	M	14	Hand tremor	>20	41
2	PD14	55	M	5	Balance	0	
2	PD17	82	M	30	Balance	8	56
2	PD23	73	M	12	Balance, Fatigue	0	41
2	PD24	53	M	8	Fatigue	2–3	
2	PD26	63	M	15	Tremor	0	10.5
2	PD28	57	M	4	Tremor	0	11.5
2	PD34	66	M	3	Dizziness, tremor	6	23
3	PD02	53	M	5	Speech, balance	2	12
3	PD04	71	M	21	Arm tremor	9	36.5
3	PD06	69	M	10	Tremor, leg weakness, sleep problems	>20	35.5
3	PD15	79	M	1	Tremor	0	7.5
3	PD18	76	M	7	Tremor, fatigue	3	8
3	PD20	74	M	4	Restless legs	0	29
3	PD25	81	M	4	Tremor	0	30
3	PD30	67	M	1	Hand tremor	0	13.5

UPDRS: Unified Parkinson’s Disease Rating Scale.

A high-resolution eye tracker (EyeLink 1,000 plus-table mounted camera) was used to measure binocular horizontal and vertical eye positions simultaneously at 0.01° spatial and 500 Hz temporal resolution (Chen et al., 2018; Kang et al., 2019). A target sticker was placed on the subject’s forehead, which allowed measurements of head movements while measuring binocular eye movements. An infrared permissive filter was used to block visible light while allowing the non-viewing eye to be tracked. Monocular calibration and validation for each eye were performed at 55 cm in the head-fixed position as described previously for all study participants (Gupta et al., 2021a). Precise calibration was achieved by using the 3-point calibration preset scheme, which allowed calibration of horizontal and vertical eye positions of each eye that subtended an angle of 0° horizontal and 10° vertical for the top target, 15° horizontal and 10° vertical for the bottom right, and 15° horizontal and 10° vertical for the bottom left as configured in EyeLink 1,000 plus. A cruciform calibration scheme was not implemented due to non-line of sight placement of the calibration scale in the case of table-mount placement of the EyeLink camera in conjunction with the vergence bar used for the presentation of LED lights. We were not able to calibrate at 30 cm using the same configuration as the camera’s view of the eyes while looking at the predefined EyeLink targets on the bottom right and bottom left was blocked by the vergence bar used for the presentation of LED lights.

For eye alignment tasks, data were obtained under binocular viewing (BV) and monocular viewing (MV) (right eye viewing and left eye viewing) conditions while looking at the target for 30 s. Out of 33 PD patients in our cohort, 7 PD subjects had their eye alignment measured at 55 cm as pilot data. After the pilot data collection, we chose the closer target viewing distance as our main interest was to evaluate eye alignment related to convergence insufficiency. Thus, the eye alignment for the remaining subjects was quantified at 30 cm. Similar to PD subjects, the controls were tested at both 55 cm and 30 cm. Convergence was measured using LED targets at 20, 55, 150, and 244 cm distances along the sagittal plane where the subjects were asked to shift their gaze from the distantly located target to near (Searle and Rowe, 2016; Kang et al., 2018; Semmlow et al., 2019, 2021).

### Data analysis

Eye movements were further analyzed with custom-written scripts in MATLAB (Mathworks, Natick, MA).

#### Fixation characteristics and eye alignment analysis

**Figure 1:**
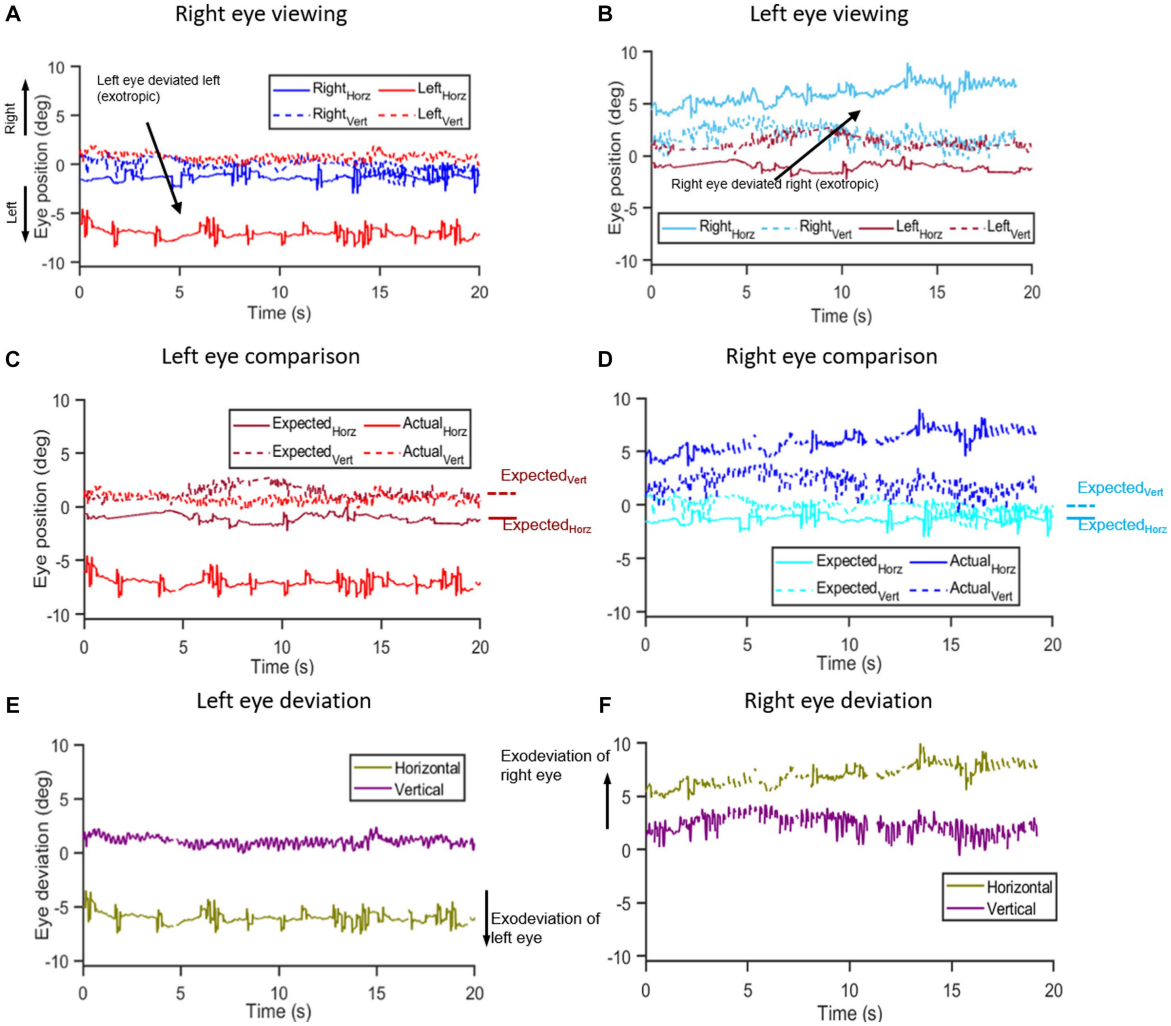
Methodology of eye deviation analysis: **(A,B)** show the time series of right and left eye horizontal and vertical eye position data from a Parkinson’s disease subject collected under right **(A)** and left **(B)** eye viewing conditions while viewing a target in primary position for 20 s. Notice that in right eye viewing condition **(A)**, the left eye is deviated to the left and during left eye viewing **(B)**, the right eye is deviated to the right indicative of an exodeviation. The viewing eye data obtained under monocular viewing is designated as the expected eye position – i.e., the right eye position data [blue trace in **(A)**] under right eye viewing is designated as right eye expected eye position data and vice versa whereas the non-viewing eye data obtained under monocular viewing is the actual eye position data, i.e., non-viewing right eye data [cyan trace in **(B)**] obtained under left eye viewing is designated as actual eye position data of the right eye and vice versa. **(C,D)** show the respective “expected” (dark blue and dark red traces) and “actual” eye position traces (light blue and light red) for the right **(C)** and left eye **(D)**. Annotations on the side show median values for expected horizontal and expected vertical eye positions to use in the calculation of the angle of deviation. The eye deviation was measured by comparing the position of each eye with itself when it was viewing vs. non-viewing (i.e., expected and actual eye positions, respectively). **(E,F)** show the computed difference in horizontal and vertical planes between the “expected” and “actual” eye positions for the right **(E)** and left eyes **(F)**.

Fixation stability was quantified by calculating the bivariate contour ellipse (BCEA) encompassing 95% of fixation points (Steinman et al., 1982; González et al., 2012; Subramanian et al., 2013). Eye position data were filtered with a moving average filter to remove fast eye movements of very low amplitude. The strabismus angles or deviation in eye alignment were hence computed based on the filtered individual eye position data obtained after the removal of fast movements in each plane. Figure 1 describes the steps undertaken to analyze eye alignment. Since the monocular calibration was performed at 55 cm, while assessing eye alignment data at 30 cm, we had to take the approach of designating *actual* and *expected* eye positions as described below. A similar approach was taken while evaluating eye alignment data at 55 cm for our initial pilot PD and control subjects.

We evaluated the eye position of the viewing eye in each monocular viewing condition to establish the empirical or baseline position to use as a reference point. Figures 1A,B depicts eye positions for monocular viewing conditions with right eye viewing (Figure 1A—right eye expected eye position data) and left eye viewing (Figure 1B—left eye expected eye position data). We computed the median value of the horizontal and vertical eye positions of the viewing eye and designated it as the *expected* eye position data (Figures 1C,D). Thus, the expected right eye position was acquired from the right eye trace under right eye viewing and the expected left eye position was derived from the left eye trace in left eye viewing. To compute the eye deviation, we defined the *actual* eye positions as follows for monocular and binocular viewing:

- Eye deviation under monocular viewing was computed by evaluating the eye position data of the non-viewing eye (*actual eye position*) and comparing it to the median expected eye position data obtained under monocular viewing.
- Eye deviation under binocular viewing: For each trial, we determined whether each eye was fixed on the target by evaluating the differences between expected and actual eye position data for each eye. Between the two eyes, the eye that deviated for a greater duration during the trial was designated as the non-fixing eye. We obtained the eye deviation under binocular viewing by computing the difference between expected median eye position data obtained under monocular viewing and actual eye position data of the non-fixing eye obtained under binocular viewing for each participant.

The 95% lower and upper bounds and the span of deviation were computed under BV and MV conditions for each subject. As a benchmark for determining normal range, we measured the 95th percentile of eye deviation in healthy control subjects recorded in our lab, which was 3.5° horizontally and 2° vertically (referred to as the threshold window below).

**Figure 2:**
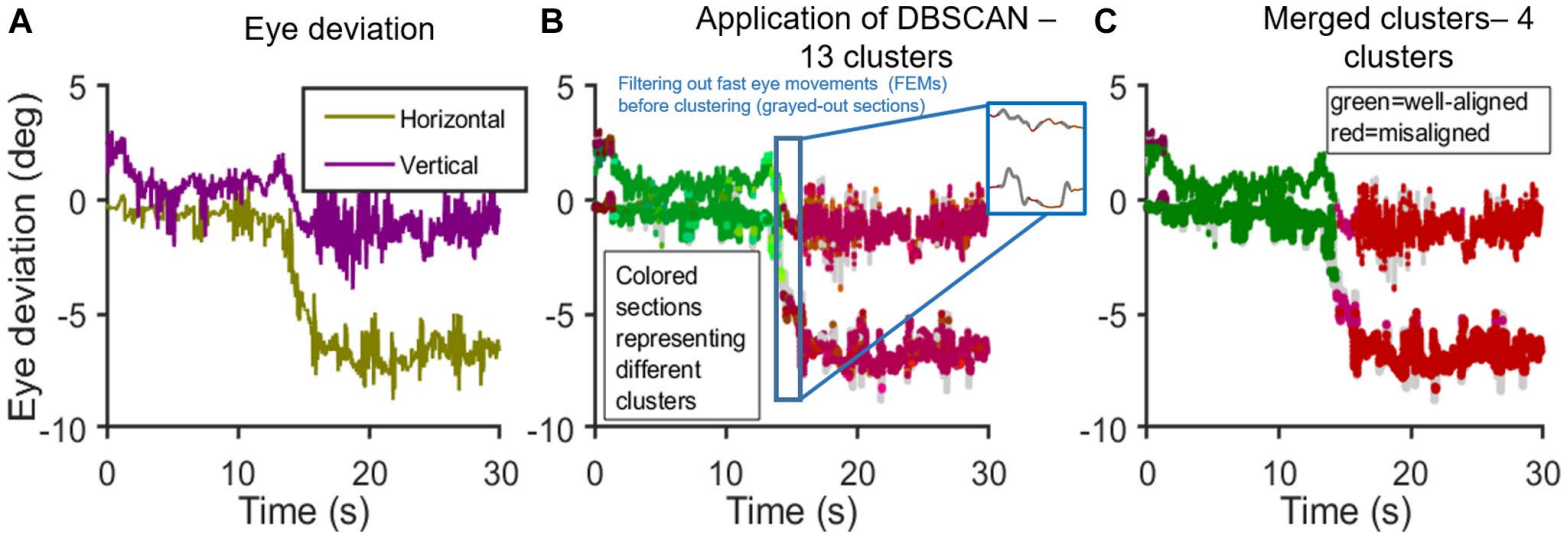
Application of DBSCAN: **(A–C)** shows the time series [x-axis: time(s); y-axis: eye deviation (deg)]; **(A)** shows the computed difference between expected and actual eye positions in horizontal and vertical planes; **(B)** shows the Initial application of DBSCAN resulting in 5 well-aligned and 8 misaligned clusters. Inset shows the instance of filtering out rapid eye movements, in this case, an SWJ; **(C)** shows a single well-aligned super-cluster. Misaligned clusters with horizontal means <2° and vertical means <1° apart are combined. End result: 1 super-cluster of good binocular alignment and 3 misaligned super-clusters.

The eye deviation (difference calculated between *expected* and *actual* eye positions) was further filtered to remove all rapid movements such as saccadic intrusions, as shown in Figure 2B (inset). The cleaned eye deviation data, devoid of the rapid movement artifact, were clustered using Density-Based Spatial Clustering and Application with Noise (DBSCAN) which allows objective assessment of control of eye deviation in intermittent strabismus as seen in PD subjects. DBSCAN uses a minimum density level estimation with a radius determined interactively to be 0.05° (epsilon) and a core point threshold potential determined adaptively to be proportional to twice the expected number of points given an even distribution of points within 95% BCEA approximation or a minimum number of points present in a verified fixational eye movement segment, whichever is greater. The clustering algorithm parameters (epsilon and minimum number of points) were chosen such that the eye trajectories arising from rapid movements with quick changes in eye alignment were not sufficiently dense to join all of the clusters together but at the same time, not setting the density threshold so high such that there were a large number of small clusters in the neighborhood. Clusters with horizontal and vertical means within the normative ranges (threshold window) were labeled as “good” or well-aligned and all others were labeled as “bad” clusters, henceforth referred to as “misaligned clusters.”

Figure 2 graphically depicts the application of DBSCAN and the clustering approach in analyzing eye deviation under binocular viewing in a PD subject. Figures 2A–C: steps in clustering time series data. Figure 2A: horizontal and vertical eye deviation obtained after taking the difference between the expected and actual eye position in horizontal and vertical planes, respectively, as outlined in Figure 1. On initial application of DBSCAN, we get 13 clusters (Figure 2B); Out of these, 5 clusters are shown in varying shades of green, when the eyes are well-aligned, and 8 clusters in varying shades of red, when the eyes are misaligned (outside the determined threshold window—3.5° horizontally and 2° vertically). Data points not part of any cluster, i.e., not density reachable from any core points, are depicted in gray. The well-aligned and misaligned clusters were further merged into super-clusters with the following criteria: 1) all well-aligned clusters combined into one super-cluster and 2) misaligned clusters with both horizontal means <2° and vertical means <1° apart were merged (Figure 2C). The total percentage of time duration when both eyes were well-aligned (within the threshold window) is referred to as “average good time%”.

The time-based weighted mean of misaligned clusters (Mean_Weighted_) was calculated to further understand the change in eye deviation under different viewing conditions. Using Eq. 1, composite values were calculated for each of the misaligned super-clusters (*n*) means (Composite_n_). Then, the time duration associated with each of these misaligned super-clusters (T*_n_*) was calculated as a fraction (FT*_n_*) of the total time (T_total_), as described in Eq. 2. Using the results from Eqs 1, 2, the cluster composite (ClusterComposite*_n_*) was calculated as per the time spent for each of the clusters as described in Eq. 3. Finally, the weighted mean of all the misaligned super-clusters was determined using Eq. (1).


(1) $$\text{Composite}=\sqrt{\left({\text{Horizontal}}^{2}+{\text{Vertical}}^{2}\right)}$$

(2) $$\begin{array}{l} {FT}_{n}=T_{n}/T_{\text{total}}\\ \left[T_{\text{total}}=\sum_{n=1}^{n=N}T_{n}\right] \end{array}$$

(3) $${\text{ClusterComposite}}_{n}={\text{Composite}}_{n}*{FT}_{n}$$

(4) $${\text{Mean}}_{\text{Weighted}}=\left(\sum {\text{ClusterComposite}}_{n}\right)/T_{\text{total}}$$

For example, as shown in Figure 2, the means of misaligned clusters were dark purple cluster [Horizontal: −0.1°; Vertical: 2.51°; Time duration: 0.69 s], pink cluster [Horizontal: −4.55°; Vertical: −0.69°; Time duration: 1.01 s], and red cluster [Horizontal: −6.76°; Vertical: −1.44° Time duration: 11.41 s]. Mean_Weighted_ of all bad clusters (Eq. 4) was found to be 6.97. Thus, the higher the value of Mean_Weighted_ suggests worse eye deviation with poor control.

#### Convergence responses

##### Fusion initiation component

*Latency:* The shift in eye position beyond 0.5° from baseline after target shift with a velocity of more than 3°/s velocity (Qing and Kapoula, 2004) was marked as vergence latency (start of *fusion initiation component*). Saccade latency was determined as the initiation time of the first conjugate saccade detected after the target jump, and the saccade detection was facilitated by Engbert’s algorithm (Engbert and Kliegl, 2003).

*Gain:* Vergence gain was determined as the ratio between the change in vergence amplitude (left eye gaze shift-right eye gaze shift) and actual vergence demand (i.e., target shift). Positive values correspond to a net convergence gain, and negative values correspond to a net divergence gain.

*Peak Velocity:* Peak velocity was measured by differentiating the fusion initiation component with respect to time.

##### Fusion maintenance component

Variance of difference in eye positions was computed from the end of the fusion initiation component when the velocity fell and stayed below 3°/s till the end of the trial, which is the *fusion maintenance component*. Micro- and macro-saccadic intrusions such as square wave jerks were excluded.

### Statistical analysis

The statistical analysis was performed in MATLAB and SPSS. Mann–Whitney U-test was used to evaluate the age of study participants between controls and PD subjects. The normality of data was evaluated using the Kolmogorov–Smirnov test. Due to normality violations, we reported the non-parametric Kruskal–Wallis H-test, and post-hoc Dunn’s multiple comparisons test for all eye alignment parameters was done for statistically significant results. Chi-square tests were used to analyze the strategies employed in response to disparity-driven vergence tasks across participant groups. Latency and fusion initiation vergence gain were analyzed across groups per the different strategies employed in response to disparity-driven vergence tasks using the Kruskal–Wallis H-test, and Dunn’s multiple comparison post-hoc tests were done for statistically significant results. Fusion maintenance/fixation position variance was analyzed across groups cumulatively for all strategies across participant groups using the Kruskal–Wallis H-test, and Dunn’s multiple comparison post-hoc tests were done for statistically significant results. The statistical significance was defined as *p* < 0.05 for all tests. The Kruskal–Wallis H-test was performed to compare disease severity *via* the Unified Parkinson’s Disease Rating Scale (UPDRS) scores across PD groups.

## Results

We recruited 33 PD patients (age: 69.16 ± 8.2 years) and 10 healthy controls (age: 65.14 ± 6.8 years) with no significant difference in age between the two groups (U = 65, *p* = 0.36). The goal of our study was to test the hypothesis that the eye misalignment, which causes diplopia, is correlated with the vergence insufficiency seen in PD. The results validating this hypothesis first outline the eye alignment during gaze holding and subsequently summarize binocular coordination in the vergence task. We applied DBSCAN to eye deviation obtained in all controls and PD subjects under MV and BV in the gaze-holding task. For control subjects, the Good Time (%) under BV was 98.3 ± 2.2. We grouped PD subjects into three groups based on the Good Time (%) under BV as follows: PD Group 1: Good Time (%) > 80% (*n* = 15); PD Group 2: Good Time (%) between 5% and 80% (*n* = 10); and PD Group 3: Good Time (%) < 5% (*n* = 8).

Here, we detail the results of the parameters of eye alignment and vergence performance of each group.

### Parameters of eye alignment (strabismus)

**Figure 3:**
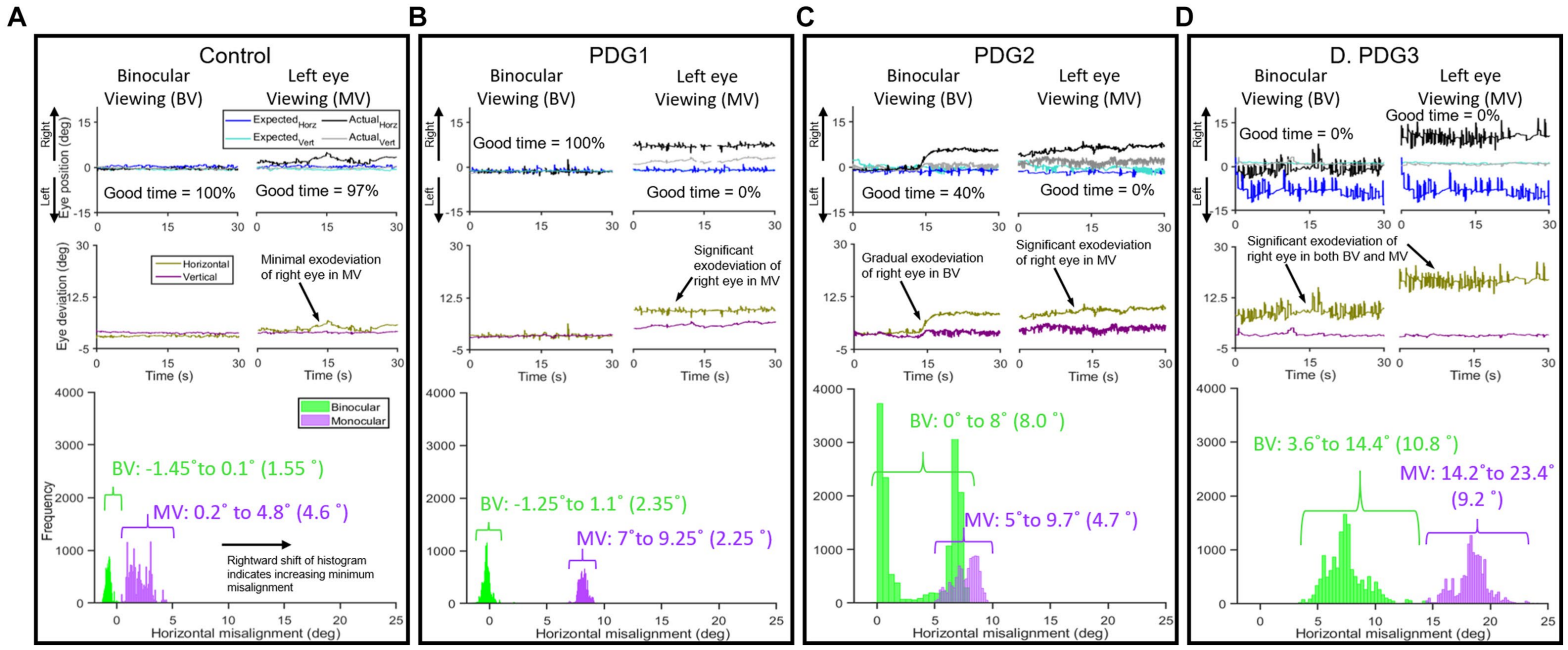
Examples of horizontal and vertical eye positions obtained from the right eye during a 30-s epoch under left eye viewing (monocular) and binocular viewing conditions: From a Control subject in BV [**(A)**-left top panel)] and MV [**(A)**-right top panel)], eye positions maintained fairly close together under BV with minimal increase in difference (exodeviation) between actual and expected eye positions observed under MV; **(A)** (middle panel): eye deviation, i.e., difference in eye positions for BV (right) and MV (left) in horizontal and vertical planes in control subject; **(A)** (bottom panel): histogram of difference of horizontal eye positions (95% lower and upper bounds with span in parenthesis) showing less spread in BV than MV with the majority of data points falling within the threshold window of 3.5°). PDG1 subject: **(B)** (left top panel): BV—eye positions maintained close together, **(B)** (right top panel): MV—significant difference (7–9.25° horizontal) between actual and expected eye position; **(B)** (middle panel): minimal difference in eye positions for BV (right) and a significantly larger difference in MV (left) in horizontal and vertical planes in PDG1; **(B)** (bottom panel): histogram of difference of horizontal eye positions showing constrained spread in BV and relatively larger spread in MV. PDG2 subject: **(C)** (left top panel): BV—eye positions maintained close together initially with right eye deviating up to 8° starting around the halfway point, **(C)** (right top panel): MV—gradually increasing rightward deviation in horizontal plane; **(C)** (middle panel): difference in eye positions for BV (right) and MV (left) in horizontal and vertical planes in PDG2 showing significantly higher deviation; **(C)** (bottom panel): histogram of difference of eye positions showing large spread in both BV and MV with increased span under BV. PDG3 subject: **(D)** (left top panel): BV—large difference in eye positions throughout, **(D)** (right top panel): MV—large angle deviation in horizontal between actual and expected eye position; **(D)** (middle panel): difference in eye positions for BV (right) and MV (left) in horizontal and vertical planes and horizontal eye deviation in PDG3; **(D)** (bottom panel): histogram of the horizontal difference of eye positions showing extremely large spread in both BV and MV with increased span under both BV and MV.

We studied the findings from the eye alignment task. Figure 3 compares the horizontal and vertical eye alignment in controls and PD Groups 1, 2, and 3. Figures 3A–D (top panel): actual and expected eye positions of the non-fixing eye under binocular viewing (BV) and of the non-viewing eye under monocular viewing (MV); (middle panel): horizontal and vertical eye deviation resulting from the angular difference in eye positions (shown in top panel), computed using actual and expected eye positions as outlined above and the histogram of horizontal eye deviation with 95% upper and lower bounds (bottom panel) with the span of eye deviation from a healthy control (Figure 3A), PD subject in Group 1 (Figure 3B), PD subject in Group 2 (Figure 3C), and PD subject in Group 3 (Figure 3D). Notice that in the control subject, the eyes are well-aligned under BV and the majority of MV (horizontal mean less than 3.5° and vertical mean less than 2°). PD subject from Group 1 (Figure 3B) had an eye alignment comparable to the healthy subject in BV (left), but with the right eye deviating out in MV (right—exophoria by ~8°). Histogram representation in Figure 3B shows comparable eye deviation upper and lower bounds and span to control subject under BV and increased horizontal eye deviation under MV. Figure 3C shows an example of a PD patient in Group 2 with an initially normal eye alignment that gradually deteriorates with the right eye drifting to the right by 8°, suggestive of exotropia, during BV (left). This patient shows increasing rightward exodeviation in monocular viewing of the right eye throughout the recording period. The histograms upper and lower bound reflect the intermittent horizontal eye deviation in BV with an increase in the span and rightward exodeviation compared to the control subject under MV. Figure 3D shows an example of a PD patient in Group 3 with increased eye misalignment of ~7°, suggestive of exotropia, during BV (left), and ~ 18° eye deviation in MV (right). The histograms upper and lower bound reflect the constant and extremely large exodeviation in BV and MV compared to the control subject. Figure 3 shows an obvious deterioration trend in spatial and temporal control of alignment as we go from PD Group 1 (similar to controls) to PD Group 3 (high degree of exodeviation).

**Figure 4:**
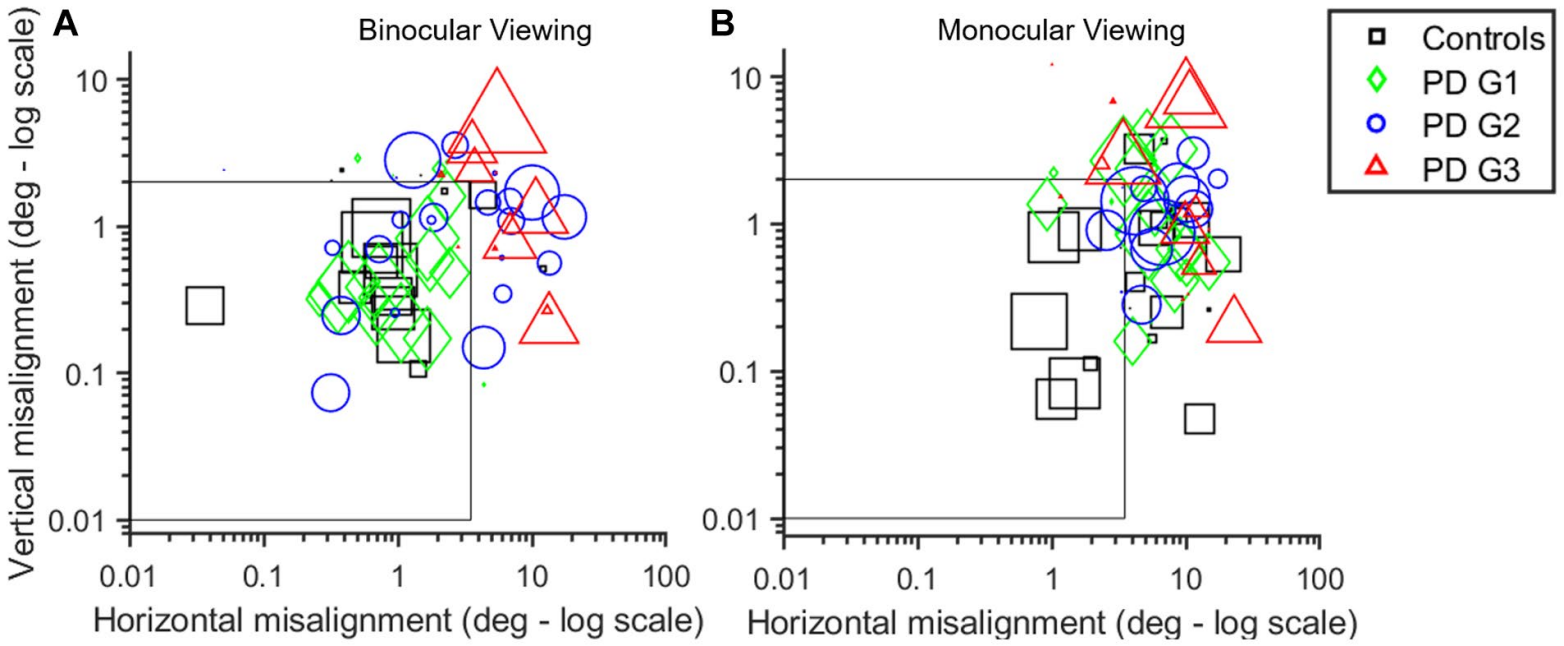
**(A,B)** Scatter plot of the log of cluster means generated using the DBSCAN algorithm for each participant in BV **(A)** and MV **(B)** in horizontal (x-axis) and vertical (y-axis) planes. Marker size is indicative of time spent in that region. Controls (black squares) are very well-controlled in BV with the majority of points within the threshold window in MV (i.e., within or near gray box depicting threshold for well-aligned clusters—3.5° horizontal; 2° vertical) than PD subjects. PDG1 subjects (green diamonds) are comparable to controls in BV but show significant deviation in MV. PDG2 subjects (blue circles) show some increase in eye deviation in BV with a large increase in eye deviation in MV. PDG3 subjects (red triangles) show significantly worse eye alignment with extremely large eye deviation reflected as the majority of red symbols being outside the threshold window, particularly in MV.

The subsequent analysis used DBSCAN clustering, as detailed in the methods section to further validate this claim. Figures 4A,B describes the mean cluster locations as identified after the application of DBSCAN and the time spent in that region for each viewing condition: BV (Figure 4A) and MV (Figure 4B). It is noteworthy that the majority of clusters belonging to controls (black squares) were within the dashed box depicting the normative threshold window calculated with expected values. PD Group1 (green diamonds) response is comparable to controls in BV but shows impairment in MV. PD Group2 (blue circles) shows a distribution of clusters both inside and outside the dashed window in BV but consistently outside the window in MV. The majority of PD Group3 (red triangles) clusters are outside of the dashed window, indicating impaired alignment in both BV and MV.

We computed the composite eye deviation for each subject under BV and right eye and left eye viewing conditions and pooled the 95% upper and lower bound histogram values and span of eye deviation of all 4 groups (Table 2). Overall, eye deviation is less in BV than in right eye (OD) and left eye (OS) viewing across all groups. PDG1 has comparable eye alignment in BV to controls but showed significantly increased eye deviation in MV. PDG2 shows increased eye deviation under both BV and MV whereas PDG3 has significantly worse eye alignment with very increased eye deviation in both BV and MV. Table 2 describes the amount of time each group spent when the eye alignment was within the threshold window (Good Time (%)) in both MV and BV. Control subjects have eyes well-aligned for significantly longer durations compared to PD patients. For all 4 groups, the eyes are well-aligned for greater periods under BV than MV. We computed the time-based weighted mean of composite eye deviation in each super-cluster (Mean_Weighted_). Table 2 depicts the Mean_Weighted_ values for misaligned clusters in controls. The values depicting eye misalignment were lowest in controls as expected and increasingly more pronounced going from PD Group 1 to PD Group 2 and finally, with the worst performance in PD Group 3. In summary, eye misalignment is commonly seen in PD patients with greater abnormalities observed under MV than in BV conditions.

**Table 2:** Eye deviation under binocular viewing and monocular viewing across 10 controls and 33 PD subjects.

Viewing condition		Parameters	Control (*n* = 10)	PDG1 (*n* = 15)	PDG2 (*n* = 10)	PDG3 (*n* = 8)	Kruskal–Wallis test *χ*^2^(df) = chi-sq, *p*-value
Both eye viewing	Eye deviation	Composite lower bound (deg)	0.05 ± 0.09	0.2 ± 0.35	0.18 ± 0.22	4.84 ± 2.56	*χ*^2^(3) = 21.08, *p* < 0.01^c^^,^^d^^,^^f^
		Composite upper bound (deg)	2.05 ± 0.76	2.63 ± 0.93	8.15 ± 5.05	10.19 ± 3.7	*χ*^2^(3) = 17.89, *p* < 0.01^b^^,^^c^^,^^e^
		Composite span (deg)	1.99 ± 0.79	2.43 ± 0.9	7.98 ± 5.13	5.36 ± 3.93	*χ*^2^(3) = 27.55, *p* < 0.01^b^^,^^c^^,^^d^^,^^e^
	Time-based control of eye deviation	Good time (%)	98.37 ± 2.29	96.39 ± 5.32	39.12 ± 19.33	10.22 ± 30.04	*χ*^2^(3) = 30.76, *p* < 0.01^b^^,^^c^^,^^d^^,^^e^
		Mean_Weighted_ (deg^2^s)	2.88 ± 1.0	1.68 ± 1.79	4.14 ± 8.12	8.74 ± 3.06	*χ*^2^(3) = 27.48, *p* < 0.01^b^^,^^c^^,^^d^^,^^e^
Left eye viewing	Eye deviation	Composite lower bound (deg)	2.64 ± 3.07	4.91 ± 5.15	4.7 ± 3.39	6.51 ± 3.65	*χ*^2^(3) = 6.35, *p* = 0.09
		Composite upper bound (deg)	4.86 ± 3.49	9.28 ± 5.08	8.08 ± 3.35	10.48 ± 4.75	*χ*^2^(3) = 7.67, *p* = 0.05
		Composite Span (deg)	2.58 ± 1.25	4.57 ± 1.19	3.68 ± 1.39	4.42 ± 1.52	*χ*^2^(3) = 12.23, *p* < 0.01^c^
	Time-based control of eye deviation	Good time (%)	48.92 ± 49.35	12.85 ± 26.08	13.97 ± 34.97	0 ± 0	*χ*^2^(3) = 9.23, *p* = 0.023^c^
		Mean_Weighted_ (deg^2^s)	3.70 ± 3.62	8.34 ± 5.04	7.98 ± 2.33	9.74 ± 4.38	*χ*^2^(3) = 10.80, *p* = 0.013^c^
Right eye viewing	Eye deviation	Composite lower bound (deg)	2.59 ± 3.29	4.46 ± 2.87	6.09 ± 4.8	5.66 ± 2.86	*χ*^2^(3) = 5.98, *p* = 0.11
		Composite upper bound (deg)	5.16 ± 4.21	8.89 ± 4.07	9.51 ± 5.37	11.15 ± 4.47	*χ*^2^(3) = 8.96, *p* = 0.03^c^
		Composite span (deg)	2.97 ± 1.16	4.6 ± 3.28	3.83 ± 2.29	6.22 ± 4.74	*χ*^2^(3) = 3.06, *p* = 0.38
	Time-based control of eye deviation	Good time (%)	50.63 ± 49.53	8.25 ± 20.38	7.96 ± 21.09	0.1 ± 0.29	*χ*^2^(3) = 9.17, *p* = 0.03^c^
		Mean_Weighted_ (deg^2^s)	3.87 ± 4.35	7.64 ± 3.39	8.57 ± 4.77	10.71 ± 5.41	*χ*^2^(3) = 10.01, *p* = 0.02^c^

(Mean ± SD); Composite lower bound: Composite minimum value of spread; Composite upper bound: Composite maximum value of spread; Average Good Time %: total percentage of time when both eyes were well-aligned; Mean_Weighted_: Time-based composite mean of bad clusters of difference of eye positions.

^a^Post-hoc Dunn’s test significance between controls and PDG1.

b Post-hoc Dunn’s test significance between controls and PDG2.

c Post-hoc Dunn’s test significance between controls and PDG3.

d Post-hoc Dunn’s test significance between PDG1 and PDG3.

e Post-hoc Dunn’s test significance between PDG1 and PDG2.

f Post-hoc Dunn’s test significance between PDG2 and PDG3.

### Parameters of vergence

**Figure 5:**
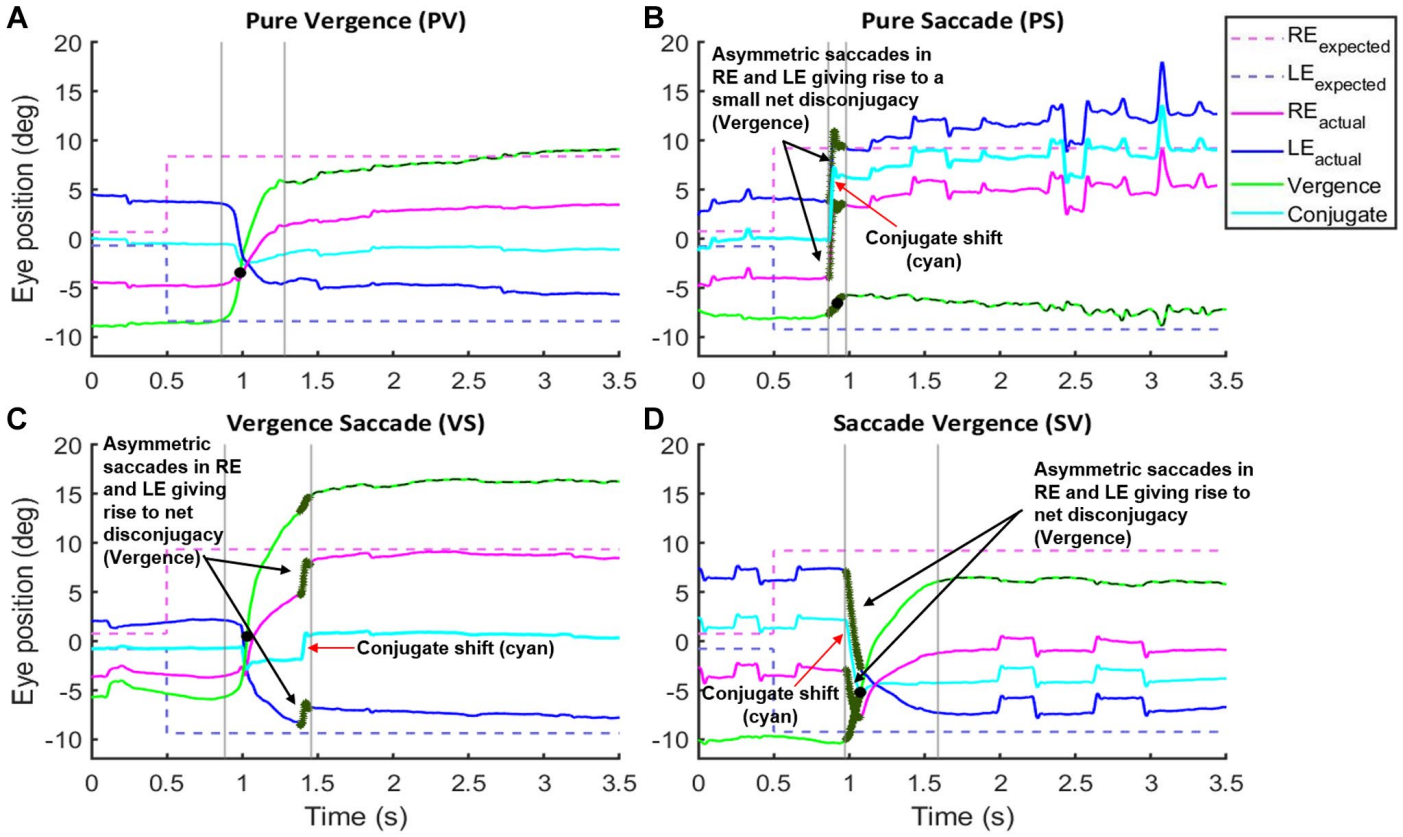
Examples of vergence initiation strategies and their distribution by group: **(A–D)** show vergence strategies seen across four groups of participants. Black solid circle denotes peak velocity. Gray lines define the start and end of fusion initiation/gaze shift. Dashed green trace defines the fusion maintenance component. Notice that in the pure vergence strategy **(A)**, the left eye moves to the right and the right eye moves to the left with a net purely disconjugate component (green trace—positive excursion suggestive of convergence response). In the pure saccade strategy **(B)**, the right and left eyes move to the right with a net conjugate movement (cyan trace) and there is a minimal change in the disconjugate component (green trace). For the vergence saccade (*VS*) strategy **(C)**, there is an initial pure vergence movement that is followed by a saccade whereas in the saccade vergence (SV) strategy **(D)**, there is a saccadic component (arrow) that precedes the pure vergence component. For the strategies incorporating conjugate shift **(B–D)** notice that the saccades are asymmetric resulting in a net disconjugate component that contributes to the vergence gain (dark green section in Re_actual_, LE_actual_, and Vergence traces emphasized with black arrows). The resultant conjugate shift (cyan trace) is emphasized with red arrows.

**Figure 6:**
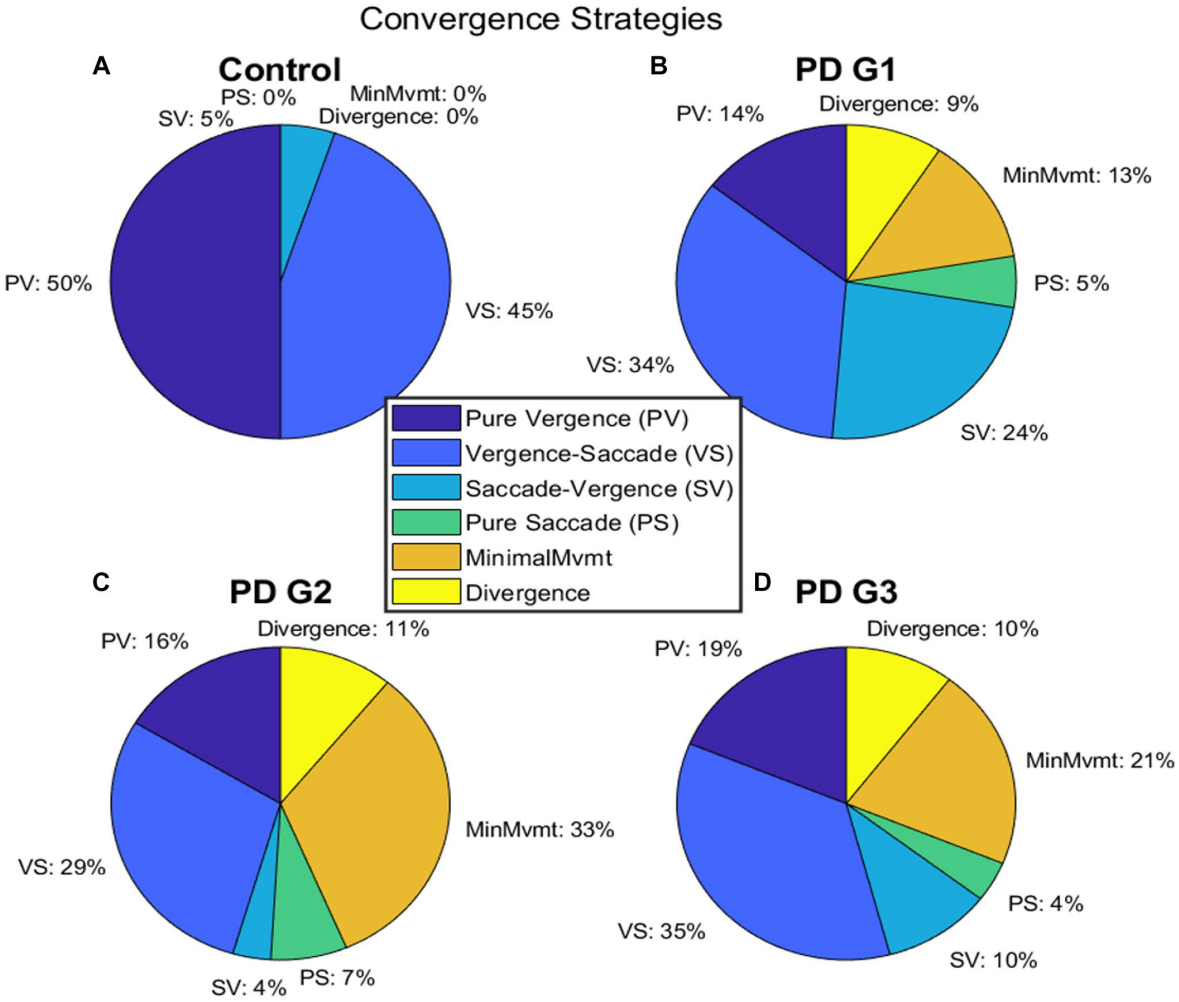
**(A–D)** depict the distribution of different strategies recruited to perform gaze shift in BV—pure vergence “PV”, vergence-saccade—“*VS*”, saccade-vergence—“SV”, and pure saccade—“PS”. In **(A)**, control subjects were successful in performing vergence-leading eye movements (PV or *VS*) 95% of the time whereas, PDG1 subjects **(B)** were successful in performing vergence-leading eye movements 44% of the time. PDG2 subjects **(C)** had significant difficulty in executing any appreciable gaze shift (33%) and could only perform vergence-leading eye movements 35% of the time. PDG3 subjects **(D)** also had significant difficulty in executing any appreciable gaze shift (21%) and executed a much higher percentage of saccade-leading eye movements 14% of the time and divergence (wrong direction) movements 10% of the time. Notice that minimal movement, wrong direction (divergence movement), and pure saccade strategies were seen only in PD subjects with a greater % of patients in PDG2 and PDG3 that exhibited minimal movement strategy.

We measured the parameters of fusion initiation and fusion maintenance vergence eye movements. We identified strategies contributing to a net convergence as shown in Figure 5. Each participant accomplished the given vergence task using different strategies. One strategy involved pure vergence (PV), i.e., binocular gaze shifts in opposite direction [pure disconjugate movement (difference between right and left eye positions = green trace) with minimal/no shift of conjugate trace (average of right and left eye position) = cyan trace)]. This strategy was highly prevalent in controls; the example is shown in Figure 5A. Figure 5B shows an example where a pure saccadic (PS) or conjugate movement was performed instead of a vergence movement. Note that the convergence trace (green, Figure 5B) has minimal shift, while each eye is making a large rapid gaze shift (i.e., saccade) in the same direction. The change in vergence (green) trace between the two vertical gray lines occurs due to an existing asymmetry between the saccades in each eye, resulting in a net disconjugate gaze shift as can be seen by comparing the amplitude of the right (blue) and left eyes (magenta) and the conjugate eye position (cyan) (Figure 5B). Figure 5C shows a leading vergence response coupled with a conjugate saccade component at the end, a strategy called vergence-saccade. In this strategy as well, the conjugate saccade component is unequal or asymmetric between the two eyes, resulting in a net disconjugacy. Figure 5D shows an initial conjugate saccadic response followed by vergence, i.e., saccade-vergence strategy (referred to as “SV”). Evaluating vergence abilities in PD patients as classified earlier, we found that approximately 13% of patients in PD Group 1, 33% in PD Group 2, and 21% in PD Group 3 were not able to make any appreciable eye movement in response to target shift in depth (Figures 6A–D). We also found that in a small percentage of trials, subjects in Group 1 (9%), Group 2 (11%), and Group 3 (10%) exhibited divergence instead of convergence. All PD groups showed significant saccadic compensations in lieu of compromised vergence movements whereas controls were successful in partial to complete convergence 95% of the time. As shown in Figures 6B–D, each PD group had a small percentage of purely saccadic movements (PS), and an asymmetry in the conjugate gaze shift in these cases led to a small net disconjugate change. There was no incidence of pure saccades in control subjects. Overall, gaze shift strategies were significantly different across the four participant groups (*χ*^2^ test, *p* = 4.97^−6^). The results indicate that vergence ability fails, and three-dimensional gaze shift relies on saccadic eye movements to compensate for vergence deficits in PD participants.

**Figure 7:**
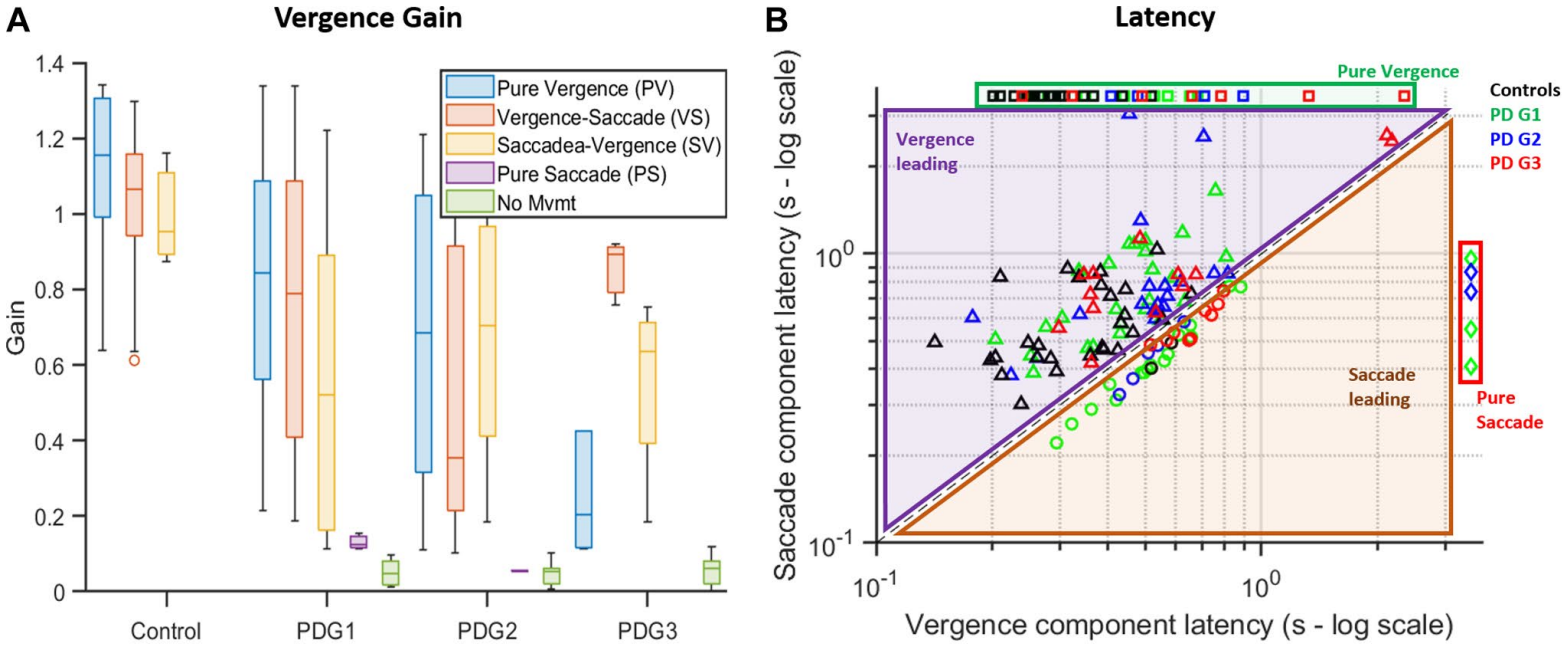
**(A)** Summary box plot of vergence gaze shift gain with respect to the strategy used. Controls have the highest overall gain compared to the PD groups across all strategies with the ability to initiate movement for all trials. PD Groups had lower overall gains and trials with pure saccades and minimal movement. Gains in conjugate segments of gaze shifts (PS, *VS*, and SV) are computed from asymmetric saccades giving rise to a net disconjugacy. **(B)** Logarithmic plot of latencies found during each trial broken down into the latency of vergence (disconjugate) movement (x-axis) and saccade (conjugate) movement (y-axis) across different groups. In the case of pure vergence (missing ordinate) or pure saccade (missing abscissa), a constant but arbitrary value was assigned in place of the missing value to plot against combination strategies (alternate strategies involving both vergence and saccade). Most controls (black squares) utilized vergence-leading strategies (PV or *VS*) (values are above the equality line). PDG1 (green diamonds), PDG2 (blue circles), and PDG3 (red triangles) subjects had several trials with saccade-leading (SV or PS) strategies.

We then evaluated gain based on the vergence strategy employed across the four groups (Figure 7A). It can be seen that net gain in controls is the highest, followed by PDG1, PDG2, and, PDG3, regardless of the strategy recruited to execute gaze shift. The subsequent analysis examined the latency of gaze shift, with respect to the strategy incorporated. Figure 7B shows latencies observed across all groups on a log scale. As shown, most gaze shifts were observed to recruit vergence-leading strategies across all four participant groups. Only PD subjects were observed to recruit saccade-leading strategies.

Table 3 describes gain and latency for pure vergence and vergence followed by the saccade strategy seen across groups. Overall, controls showed near-perfect convergence whereas all PD groups showed compromised vergence responses with reduced vergence gain for both strategies.

**Table 3:** Convergence parameters: descriptive statistics (*n* = 33).

Fusion initiation parameters	Control (*n* = 10)	PDG1 (*n* = 15)	PDG2 (*n* = 10)	PDG3 (*n* = 8)	Kruskal–Wallis
Pure vergence movements only					
Fusion-initiating vergence gain	1.11 ± 0.2	0.81 ± 0.4	0.67 ± 0.42	0.33 ± 0.38	*χ*^2^(3) = 16.02, *p* < 0.01^b^^,^^c^^,^^d^
Fusion-initiating vergence latency	0.28 ± 0.07	0.47 ± 0.17	0.49 ± 0.23	0.88 ± 0.74	*χ*^2^(3) = 14.10, *p* < 0.01^c^^,^^d^
Vergence followed by saccadic movements					
Fusion-initiating vergence gain	1.04 ± 0.07	0.78 ± 0.39	0.53 ± 0.42	0.86 ± 0.09	*χ*^2^(3) = 16.68, *p* < 0.01^b^
Fusion-initiating vergence latency	0.34 ± 0.12	0.64 ± 0.29	0.51 ± 0.67	0.72 ± 0.69	*χ*^2^(3) = 37.22, *p* < 0.01^b^^,^^e^
Fusion maintenance					
Fusion maintenance vergence variance	0.3 ± 0.32	0.37 ± 0.59	0.38 ± 0.24	1.75 ± 3.39	*χ*^2^(3) = 9.60, *p* = 0.022^c^^,^^d^^,^^f^

Vergence Gain: Actual disconjugate component during dynamic gaze shift/Expected disconjugate component per target displacement; Vergence latency: Time to gaze shift initiation; Fusion maintenance variance: Variance in angle between two eyes at the end of dynamic vergence gaze shift, when eyes are holding still.

^a^Post-hoc Dunn’s test significance between controls and PDG1.

b Post-hoc Dunn’s test significance between controls and PDG2.

c Post-hoc Dunn’s test significance between controls and PDG3.

d Post-hoc Dunn’s test significance between PDG1 and PDG3.

e Post-hoc Dunn’s test significance between PDG1 and PDG2.

f Post-hoc Dunn’s test significance between PDG2 and PDG3.

Table 3 describes strategy-specific latencies across groups. Overall, controls had the lowest latencies followed by PD Group 1, Group 2, and Group 3, regardless of the strategy recruited to execute the gaze shift.

Finally, for the fusion maintenance phase, the variance of eye position difference between two eyes after dynamic vergence gaze shift, when eyes are holding still, was calculated. Table 3 summarizes the variance trend seen across the four participant groups. Overall, subjects in PD Group 3 had higher deficits in fusion-sustaining components in comparison to PD Groups 1 and 2 and controls, implying a decreasing ability to maintain fusion after convergence as a result of PD.

No significant correlation was found between disease severity (UPDRS) and eye alignment and vergence deficits with mean UPDRS values for the three PD groups found to be: Group 1 = 33.69 ± 14.22, Group 2 = 38.43 ± 22.61, and Group 3 = 23.44 ± 1, (*p* > 0.05).

Our results successfully showed that overall, participants who had marked convergence insufficiency also had a relatively worse eye alignment and participants with relatively good eye alignment had compromised but milder convergence deficits. However, binocular dysfunction might not be correlated with the standard Parkinson’s disease severity scale.

## Discussion

Oculomotor abnormalities are frequently seen in PD (Repka et al., 1996; Biousse et al., 2004; Almer et al., 2012; Anderson and MacAskill, 2013; Kang et al., 2018; Borm et al., 2020). The majority of the studies describe saccadic and gaze-holding abnormalities in PD. These include increased frequency and amplitude of saccadic intrusions and square wave jerks (Averbuch-Heller et al., 1999; Leigh and Riley, 2000; Beh et al., 2017; Otero-Millan et al., 2018; Beylergil et al., 2022). Patients with PD have increased saccadic latencies, hypometric, slow and staircase saccades, and more frequent errors during anti-saccade tasks (Zee et al., 1992; Antoniades et al., 2015; Ghasia and Shaikh, 2015; Shaikh and Ghasia, 2017, 2019; Otero-Millan et al., 2018; Beylergil et al., 2022). Eye movement abnormalities affecting binocular alignment (strabismus) and vergence deficits are not uncommon in PD (Hanuška et al., 2015; Kang et al., 2018; Borm et al., 2020; Smilowska et al., 2020; Gupta et al., 2021a). Clinical studies have speculated a causal relationship between vergence insufficiency and strabismus in PD with variable reports of improvement of deficits with medical treatment (Racette et al., 1999; Almer et al., 2012; Kang et al., 2018). Recent studies have increasingly recognized that diplopia is common in PD ranging from 20 to 30% (Schindlbeck et al., 2017; Visser et al., 2019; Smilowska et al., 2020; Santos-García et al., 2021). There is some evidence to support that the presence of diplopia could be associated with cognitive decline and apathy and a worse prognosis (Santos-García et al., 2010; Naumann et al., 2021). It is also increasingly recognized that diplopia in PD is under-reported and under-recognized as the majority of studies use self-reported questionnaires to study the prevalence of diplopia (Ekker et al., 2017). In our study, all PD patients exhibited increased near point of convergence (NPC > 10 cm). Out of 33 patients, only 2 had no measurable strabismus or eye deviation based on the results of the ophthalmological examination; however, both of those PD patients also had eye deviation under monocular viewing conditions along with reduced convergence gain and prolonged latency of gaze shift compared to controls.

Strabismus or eye misalignment interferes with visual-motor coordination of eye movements, resulting in disconjugate and cross-axis eye movements during visually guided saccades, variable and subnormal vergence responses, and fixation instability (Bucci et al., 1997; Kapoula et al., 1997; Fu et al., 2007; Ghasia et al., 2015, 2018). In our study, eye movement recordings were used to quantify time-based control of eye alignment under binocular viewing. Vergence is elicited either by retinal disparity or accommodative blur. When one eye is occluded under monocular viewing, the occluded eye position can decay and become heterophoric. Heterophoria is dependent upon accommodative convergence (SCHROEDER et al., 1996; Lee et al., 2009). We found that more than 50% of PD subjects had intermediate to poor control of eye alignment under binocular viewing and all of them had some increase in exodeviation under monocular viewing (exophoria). At first glance, this might indicate that reduced convergence abilities further hinder monocular control and increase misalignment under monocular viewing, since the accommodation (blur-driven) circuit is cross-coupled with the vergence (retinal disparity-driven) circuit (Gupta et al., 2021a). However, it is equally probable that the inherent retinal disparity cue (binocular viewing) allows for better control of eye alignment which when taken away results in poor monocular control (Horwood and Riddell, 2014; Ooi and He, 2015). This perspective is further supported by our data, in that control subjects, who do not have convergence insufficiency, still exhibit worse eye alignment in monocular viewing as compared to binocular viewing conditions. Only a handful of studies have quantified vergence in PD and have reported increased latency with variable effects on convergence velocity and gain (Hanuška et al., 2015; Gupta et al., 2021a,b). Our vergence results revealed a similar overall trend, and controls had higher vergence gain and lower latencies than PD patients. PD Group 1 showed a greater frequency of vergence-leading strategies and lower saccadic compensation along with higher vergence gain and lower latencies than Groups 2 and 3. PD subjects with good eye alignment under binocular viewing were less likely to exhibit saccadic responses to disparity-driven vergence, whereas those with poor control and worse eye alignment under binocular viewing were more likely to exhibit saccadic responses or had minimal eye movement responses to pure symmetrical step stimuli located at different initial vergence angle. PD Group 3 patients who exhibited pure vergence or combined vergence/saccade responses to disparity-driven vergence exhibited deficits of both fusion-initiating (prolonged latencies, reduced gain, and peak vergence velocities) and fusion-maintaining components (increased eye position variance). We also found that PD subjects can have abnormalities in convergence such as prolonged latencies and reduced gain despite having relatively good eye alignment.

There was no correlation between disease severity and eye alignment abnormalities. The majority of the studies evaluating diplopia in PD are based on results obtained from subjective self-reported questionnaires (Davidsdottir et al., 2005; Borm et al., 2020; Naumann et al., 2021; Santos-García et al., 2021; van der Lijn et al., 2022). PD subjects can have CI without symptoms of diplopia *per se*; thus, binocular dysfunction is frequently overlooked (Lepore, 2006; Sauerbier and Ray Chaudhuri, 2013; Ekker et al., 2017). A handful of studies have evaluated convergence insufficiency and eye alignment in PD using standard ophthalmic clinical examination. These studies have found no differences between the prevalence of CI and the duration of disease, with patients with early PD also likely to have CI. Furthermore, no systematic differences were observed between disease severity and extent of exodeviation at near or convergence insufficiency (Repka et al., 1996; Biousse et al., 2004; Almer et al., 2012; Visser et al., 2019). We have used eye movement recordings to quantify binocular dysfunction in PD including time-based variability of eye deviation and vergence abnormalities. It allows quantification of the control of eye deviation over time and quantification of latencies, gain of vergence, and ability to maintain eyes on the target at the end of vergence. These parameters cannot be precisely quantified or are limited by subjectivity in measurements when evaluated by standard clinical exam techniques alone. Quantification of these parameters would be critical particularly while evaluating treatment outcomes.

Non-human primate (NHP) studies have provided important insights into the neural correlates of oculomotor abnormalities observed in strabismus. Cells within the rostral superior colliculus have been shown to modulate vergence (Jiang, 1996; Chaturvedi and Van Gisbergen, 1999, 2000; Krauzlis, 2003; Van Horn et al., 2013; Pallus et al., 2018). Electrical micro-stimulation of the superior colliculus in strabismic NHPs has been shown to evoke disconjugate saccades (both in direction and amplitude) (Fleuriet et al., 2016). The cells within the rostral superior colliculus have also been shown to carry signals related to horizontal eye misalignment and fixation preference in strabismic NHPs (Van Horn et al., 2013; Upadhyaya et al., 2017; Upadhyaya and Das, 2019). The midbrain houses neurons critical to controlling the vergence position (Judge and Cumming, 1986; Shook et al., 1990; Buttner-Ennever, 2006) and velocity (May et al., 1992) in the mesencephalic reticular formation, in a region called the supra-oculomotor area (SOA). Neurons within the SOA, encoding vergence responses in normal animals, were found to encode horizontal misalignment in strabismic monkeys (Das, 2011, 2012; Walton et al., 2017). The SOA receives inputs from the deep cerebellar nuclei (fastigial nucleus and interpositus nucleus) which in turn project to the ocular motor neurons innervating slow extraocular muscle fibers (Mays, 1984; Judge and Cumming, 1986; Stanton et al., 1988).

PD is a progressive neurodegenerative disorder caused due to degeneration of dopaminergic projections between the substantia nigra pars compacta (SNc) and the remaining basal ganglia circuitry. There is increasing evidence that the subthalamic nucleus, one of the key regions demonstrating abnormal neural discharge in PD, has visuo-oculomotor neurons (Matsumura et al., 1992). The eye movement cells in monkeys and human PD subjects were primarily in the ventral portion of the subthalamic nucleus (STN) and comprised 20% of the share (Matsumura et al., 1992; Hikosaka et al., 2000; Fawcett et al., 2005, 2007; Sieger et al., 2013). They receive inputs from the prefrontal association cortex (Monakow et al., 1978), the frontal eye field (FEF) (Stanton et al., 1988; Huerta and Kaas, 1990), or the supplementary eye field (SEF) (Huerta and Kaas, 1990). Human and non-human primate physiologic and anatomic studies suggest that STN plays a role in processing information related to eye movements (Matsumura et al., 1992; Hikosaka et al., 2000). The oculomotor neurons in STN in PD patients showed task-specific responses to cued versus self-paced saccades and responded to both passive limb movement and voluntary eye movement and scanning eye movements. There is increasing anatomic and neurophysiologic evidence that STN is connected with brainstem and cerebellar circuitry, particularly areas intricately involved with driving vergence and strabismus. Two possible pathways by which STN could modulate vergence are as follows: The first pathway involves the substantia nigra pars reticulata (SNr)—one of the output stations of the basal ganglia (Hopkins and Niessen, 1976; Jayaraman et al., 1977; Graybiel, 1978). SNr receives inputs from the STN and projects to the SOA *via* superior colliculus (Shaikh et al., 2004). Thus, impairment in SNr output as seen in PD could affect vergence. The second pathway directly involves the cerebellum (Hill et al., 2013). This pathway involves subthalamo-ponto-cerebellar projections to the deep cerebellar nuclei *via* precerebellar pontine nuclei (Jenkinson et al., 2009). Further downstream from the subthalamo-ponto-cerebellar projections, the cerebello-pontine fibers connect the deep cerebellar nuclei to the strabismus angle-sensitive neurons in SOA (Das, 2011, 2012; Joshi and Das, 2013).

Debates exist in the literature concerning the neural control of binocular coordination of eye movements (Ramat et al., 2006; Cullen and Van Horn, 2011; King, 2011). Previous studies have demonstrated complex, non-linear interactions between the saccade (conjugate) and vergence (disconjugate) subsystems (Zee et al., 1992; van Leeuwen et al., 1998). Although saccadic and vergence eye movements are thought to be generated by different neural subsystems, the occurrence of a saccade during a vergence movement increases vergence velocity (Enright, 1984). In addition, Collewijin et al. have shown that for saccades that occur during vergence, the peak velocity of the saccades is reduced and the saccadic duration is increased (Collewijn et al., 1995). Studies have shown that the crux of the interaction between saccades and vergence movements is within the omnipause neurons (OPNs). Electrical micro-stimulation of the OPNs has been shown to increase the tonic inhibition of vergence and saccadic velocity neurons slowing both the vergence and saccade components (Zee and Levi, 1989; Ramat et al., 2005). Thus, the release of OPN inhibition during a saccade allows the vergence velocity neurons to fire more vigorously, thereby increasing the vergence velocity. The motor neurons receive both saccadic pulse-step signals and the vergence velocity and vergence position signals. Thus, the occurrence of a horizontal saccade during a vergence movement results in combination of the vergence and saccadic signals producing a net pulse-step signal which is larger in one eye than the other resulting in saccades of unequal size.

Our results demonstrate that the vergence abnormalities in PD subjects systematically correlate with both the angle and control of eye alignment. The target stimuli within our study were pure vergence stimuli with no retinal stimulation to the saccadic system. Yet, saccades were observed, especially within the slower vergence responses. We speculate, based upon the neurophysiology studies, that the responses to symmetrical vergence stimuli (along the midline) will evoke the near response cells (Mays et al., 1991; Zhang et al., 1992), and when the vergence velocity is below a preferred threshold, a saccade may be initiated by (1) the excitation of burst neurons, (2) the inhibition of OPNs, or (3) both the excitation and inhibition of burst neurons and OPNs. Our data support an interaction exists between the vergence and saccade subsystems in PD patients. Thus, convergence abnormalities in PD may be the result of the direct effects of the disease on vergence motor control, coupled with disturbances in the saccadic pathway. Fast vergence eye movements are disrupted in PD which comprise both convergence and divergence abnormalities (Hanuška et al., 2015; Gupta et al., 2021a,b). Future studies evaluating whether divergence insufficiency is associated with esotropia or esophoria in PD will provide a deeper understanding of issues related to vergence abnormalities and eye misalignment in Parkinson’s disease. Future neurophysiology and behavioral studies are needed to further understand the interaction between saccade and vergence eye movements.

Our PD patient cohort was referred by neurologists, based on their PD diagnosis. On further ophthalmological examination and eye movement recordings, it was discovered that all of them had some binocular dysfunction deficit, implying that these are severely under-reported or under-recognized. In addition, similar to previous studies, we found that eye deviation and vergence abnormalities are frequently seen in PD subjects with no systematic correlation with the severity of PD (Wu et al., 2018; Alhassan et al., 2020). Video-oculography was used to quantify eye movements and report deficits that are frequently not recognized with standard clinical assessments, for example, variability of the angle of ocular misalignment under different viewing conditions or precise measures of vergence abnormalities including latencies and saccadic compensation seen in neurodegenerative disorders (Gupta et al., 2021a,b). Thus, our study describes objective measures to evaluate vergence abnormalities and quantify eye deviation in PD parameters that may be of interest to assess treatment response. Further studies on early-stage and pre-symptomatic PD subjects are also needed to determine whether vergence abnormalities and strabismus can serve as a biomarker of the disease.

## Data availability statement

The raw data supporting the conclusions of this article will be made available by the authors, without undue reservation.

## Ethics statement

The studies involving humans were approved by Cleveland Clinic Institutional Review Board. The studies were conducted in accordance with the local legislation and institutional requirements. The participants provided their written informed consent to participate in this study.

## Author contributions

PG, AS, and FG: study design and manuscript drafting. PG, JM, JJ, AS, and FG: experiment design. PG, SB, AS, CK, and FG: data acquisition. PG and FG: data analysis and statistical analysis. PG, JM, SB, JJ, CK, AS, and FG: manuscript editing. All authors contributed to the article and approved the submitted version.

## References

[ref1] Alhassan M. Hovis J. K. Almeida Q. J. (2020). Stereopsis and ocular alignment in Parkinson’s disease patients with and without freezing of gait symptoms. Clin. Exp. Optom. 103, 513–519. doi: 10.1111/cxo.12961, PMID: 31441118

[ref2] Almer Z. Klein K. S. Marsh L. Gerstenhaber M. Repka M. X. (2012). Ocular motor and sensory function in Parkinson’s disease. Ophthalmology 119, 178–182. doi: 10.1016/j.ophtha.2011.06.040, PMID: 21959370 PMC3251710

[ref3] Anderson T. J. MacAskill M. R. (2013). Eye movements in patients with neurodegenerative disorders. Nat. Rev. Neurol. 9, 74–85. doi: 10.1038/nrneurol.2012.273 23338283

[ref4] Antoniades C. A. Demeyere N. Kennard C. Humphreys G. W. Hu M. T. (2015). Antisaccades and executive dysfunction in early drug-naive Parkinson’s disease: the discovery study. Mov. Disord. 30, 843–847. doi: 10.1002/mds.26134, PMID: 25600361

[ref5] Armstrong R. A. (2015). Oculo-visual dysfunction in Parkinson’s disease. J. Parkinsons Dis. 5, 715–726. doi: 10.3233/JPD-150686, PMID: 26599301 PMC4927837

[ref6] Averbuch-Heller L. Stahl J. S. Hlavin M. L. Leigh R. J. (1999). Square-wave jerks induced by pallidotomy in parkinsonian patients. Neurology 52, 185–188. doi: 10.1212/WNL.52.1.185, PMID: 9921873

[ref7] Beh S. C. Frohman T. C. Frohman E. M. (2017). Cerebellar control of eye movements. J. Neuroophthalmol. 37, 87–98. doi: 10.1097/WNO.0000000000000456 27643747

[ref8] Beylergil S. B. Murray J. Noecker A. M. Gupta P. Kilbane C. McIntyre C. C.. (2022). Temporal patterns of spontaneous fixational eye movements: the influence of basal ganglia. J. Neuroophthalmol. 42, 45–55. doi: 10.1097/WNO.0000000000001452, PMID: 34812763

[ref9] Biousse V. Skibell B. C. Watts R. L. Loupe D. N. Drews-Botsch C. Newman N. J. (2004). Ophthalmologic features of Parkinson’s disease. Neurology 62, 177–180. doi: 10.1212/01.WNL.0000103444.45882.D8 14745050

[ref10] Borm C. Visser F. Werkmann M. de Graaf D. Putz D. Seppi K.. (2020). Seeing ophthalmologic problems in Parkinson disease: results of a visual impairment questionnaire. Neurology 94, e1539–e1547. doi: 10.1212/WNL.0000000000009214, PMID: 32161030 PMC7251522

[ref11] Bucci M. P. Kapoula Z. Eggert T. Garraud L. (1997). Deficiency of adaptive control of the binocular coordination of saccades in strabismus. Vis. Res. 37, 2767–2777. doi: 10.1016/S0042-6989(97)00093-X, PMID: 9373675

[ref12] Buttner-Ennever J. A. (2006). The extraocular motor nuclei: organization and functional neuroanatomy. Prog. Brain Res. 151, 95–125. doi: 10.1016/S0079-6123(05)51004-5 16221587

[ref13] Chaturvedi V. van Gisbergen J. A. (1999). Perturbation of combined saccade-vergence movements by microstimulation in monkey superior colliculus. J. Neurophysiol. 81, 2279–2296. doi: 10.1152/jn.1999.81.5.2279, PMID: 10322066

[ref14] Chaturvedi V. Van Gisbergen J. A. (2000). Stimulation in the rostral pole of monkey superior colliculus: effects on vergence eye movements. Exp. Brain Res. 132, 72–78. doi: 10.1007/s002219900221, PMID: 10836637

[ref15] Chen D. Otero-Millan J. Kumar P. Shaikh A. G. Ghasia F. F. (2018). Visual search in amblyopia: abnormal fixational eye movements and suboptimal sampling strategies. Invest. Ophthalmol. Vis. Sci. 59, 4506–4517. doi: 10.1167/iovs.18-24794, PMID: 30208418

[ref16] Collewijn H. Erkelens C. J. Steinman R. M. (1995). Voluntary binocular gaze-shifts in the plane of regard: dynamics of version and vergence. Vis. Res. 35, 3335–3358. doi: 10.1016/0042-6989(95)00082-P, PMID: 8560804

[ref17] Cullen K. E. Van Horn M. R. (2011). The neural control of fast vs. slow vergence eye movements. Eur. J. Neurosci. 33, 2147–2154. doi: 10.1111/j.1460-9568.2011.07692.x, PMID: 21645108

[ref18] Das V. E. (2011). Cells in the supraoculomotor area in monkeys with strabismus show activity related to the strabismus angle. Ann. N. Y. Acad. Sci. 1233, 85–90. doi: 10.1111/j.1749-6632.2011.06146.x, PMID: 21950980 PMC3285100

[ref19] Das V. E. (2012). Responses of cells in the midbrain near-response area in monkeys with strabismus. Invest. Ophthalmol. Vis. Sci. 53, 3858–3864. doi: 10.1167/iovs.11-9145, PMID: 22562519 PMC3390217

[ref20] Davidsdottir S. Cronin-Golomb A. Lee A. (2005). Visual and spatial symptoms in Parkinson’s disease. Vis. Res. 45, 1285–1296. doi: 10.1016/j.visres.2004.11.006 15733961

[ref21] Ekker M. S. Janssen S. Seppi K. Poewe W. de Vries N. M. Theelen T.. (2017). Ocular and visual disorders in Parkinson’s disease: common but frequently overlooked. Parkinsonism Relat. Disord. 40, 1–10. doi: 10.1016/j.parkreldis.2017.02.014, PMID: 28284903

[ref22] Engbert R. Kliegl R. (2003). Microsaccades uncover the orientation of covert attention. Vis. Res. 43, 1035–1045. doi: 10.1016/S0042-6989(03)00084-1 12676246

[ref23] Enright J. T. (1984). Changes in vergence mediated by saccades. J. Physiol. 350, 9–31. doi: 10.1113/jphysiol.1984.sp015186, PMID: 6747862 PMC1199254

[ref24] Fawcett A. P. Cunic D. Hamani C. Hodaie M. Lozano A. M. Chen R.. (2007). Saccade-related potentials recorded from human subthalamic nucleus. Clin. Neurophysiol. 118, 155–163. doi: 10.1016/j.clinph.2006.09.016, PMID: 17097341

[ref25] Fawcett A. P. Dostrovsky J. O. Lozano A. M. Hutchison W. D. (2005). Eye movement-related responses of neurons in human subthalamic nucleus. Exp. Brain Res. 162, 357–365. doi: 10.1007/s00221-004-2184-7, PMID: 15599721

[ref26] Fleuriet J. Walton M. M. G. Ono S. Mustari M. J. (2016). Electrical microstimulation of the superior colliculus in strabismic monkeys. Invest. Ophthalmol. Vis. Sci. 57, 3168–3180. doi: 10.1167/iovs.16-19488, PMID: 27309621 PMC4928695

[ref27] Fu L. Tusa R. J. Mustari M. J. das V. E. (2007). Horizontal saccade disconjugacy in strabismic monkeys. Invest. Ophthalmol. Vis. Sci. 48, 3107–3114. doi: 10.1167/iovs.06-0955, PMID: 17591880 PMC2562538

[ref28] Ghasia F. F. Otero-Millan J. Shaikh A. G. (2018). Abnormal fixational eye movements in strabismus. Br. J. Ophthalmol. 102, 253–259. doi: 10.1136/bjophthalmol-2017-310346 28698242

[ref29] Ghasia F. F. Shaikh A. G. (2015). Experimental tests of hypotheses for microsaccade generation. Exp. Brain Res. 233, 1089–1095. doi: 10.1007/s00221-014-4188-2, PMID: 25563497

[ref30] Ghasia F. F. Shaikh A. G. Jacobs J. Walker M. F. (2015). Cross-coupled eye movement supports neural origin of pattern strabismus. Invest. Ophthalmol. Vis. Sci. 56, 2855–2866. doi: 10.1167/iovs.15-16371 26024072 PMC4419776

[ref31] González E. G. Wong A. M. F. Niechwiej-Szwedo E. Tarita-Nistor L. Steinbach M. J. (2012). Eye position stability in amblyopia and in normal binocular vision. Invest. Ophthalmol. Vis. Sci. 53, 5386–5394. doi: 10.1167/iovs.12-9941, PMID: 22789926

[ref32] Graybiel A. M. (1978). Organization of the nigrotectal connection: an experimental tracer study in the cat. Brain Res. 143, 339–348. doi: 10.1016/0006-8993(78)90573-5, PMID: 630412

[ref33] Gupta P. Beylergil S. Murray J. Jacobs J. Kilbane C. Shaikh A. G.. (2021a). Effects of Parkinson disease on blur-driven and disparity-driven vergence eye movements. J. Neuroophthalmol. 41, 442–451. doi: 10.1097/WNO.0000000000001422, PMID: 34788236

[ref34] Gupta P. Beylergil S. Murray J. Kilbane C. Ghasia F. F. Shaikh A. G. (2021b). Computational models to delineate 3D gaze-shift strategies in Parkinson’s disease. J. Neural Eng. 18:0460a5. doi: 10.1088/1741-2552/ac123e, PMID: 34233315 PMC8863489

[ref35] Hamedani A. G. Maguire M. G. Marras C. Willis A. W. (2021). Prevalence and risk factors for double vision in Parkinson disease. Mov. Disord. Clin. Pract. 8, 709–712. doi: 10.1002/mdc3.13220, PMID: 34307743 PMC8287164

[ref36] Hanuška J. Bonnet C. Rusz J. Sieger T. Jech R. Rivaud-Péchoux S.. (2015). Fast vergence eye movements are disrupted in Parkinson’s disease: a video-oculography study. Parkinsonism Relat. Disord. 21, 797–799. doi: 10.1016/j.parkreldis.2015.04.014, PMID: 25935708

[ref37] Hikosaka O. Takikawa Y. Kawagoe R. (2000). Role of the basal ganglia in the control of purposive saccadic eye movements. Physiol. Rev. 80, 953–978. doi: 10.1152/physrev.2000.80.3.953, PMID: 10893428

[ref38] Hill K. K. Campbell M. C. McNeely M. E. Karimi M. Ushe M. Tabbal S. D.. (2013). Cerebral blood flow responses to dorsal and ventral STN DBS correlate with gait and balance responses in Parkinson’s disease. Exp. Neurol. 241, 105–112. doi: 10.1016/j.expneurol.2012.12.003, PMID: 23262122 PMC3570746

[ref39] Hopkins D. A. Niessen L. W. (1976). Substantia Nigra projections to the reticular formation, superior colliculus and central gray in the rat, cat and monkey. Neurosci. Lett. 2, 253–259. doi: 10.1016/0304-3940(76)90156-7, PMID: 19604767

[ref40] Horwood A. M. Riddell P. M. (2014). Disparity-driven vs blur-driven models of accommodation and convergence in binocular vision and intermittent strabismus. J. AAPOS 18, 576–583. doi: 10.1016/j.jaapos.2014.08.009, PMID: 25498466 PMC4270963

[ref41] Huerta M. F. Kaas J. H. (1990). Supplementary eye field as defined by intracortical microstimulation: connections in macaques. J. Comp. Neurol. 293, 299–330. doi: 10.1002/cne.902930211, PMID: 19189718

[ref42] Jayaraman A Batton R. Carpenter MB., Nigrotectal projections in the monkey: an autoradiographic study. Brain Res. (1977). 135:: p. 147–152, doi: 10.1016/0006-8993(77)91058-7, PMID: 410480

[ref43] Jenkinson N. Nandi D. Muthusamy K. Ray N. J. Gregory R. Stein J. F.. (2009). Anatomy, physiology, and pathophysiology of the pedunculopontine nucleus. Mov. Disord. 24, 319–328. doi: 10.1002/mds.22189, PMID: 19097193

[ref44] Jiang H. (1996). Near-response-related neural activity in the rostral superior colliculus of the cat. Soc. Neurosci. Abstr. 22:662.

[ref45] Joshi A. C. Das V. E. (2013). Muscimol inactivation of caudal fastigial nucleus and posterior interposed nucleus in monkeys with strabismus. J. Neurophysiol. 110, 1882–1891. doi: 10.1152/jn.00233.2013, PMID: 23883862 PMC3798947

[ref46] Judge S. J. Cumming B. G. (1986). Neurons in the monkey midbrain with activity related to vergence eye movement and accommodation. J. Neurophysiol. 55, 915–930. doi: 10.1152/jn.1986.55.5.915, PMID: 3711972

[ref47] Kang S. L. Beylergil S. B. Otero-Millan J. Shaikh A. G. Ghasia F. F. (2019). Fixational eye movement waveforms in amblyopia: characteristics of fast and slow eye movements. J. Eye Mov. Res. 12:10.16910/jemr.12.6.9. doi: 10.16910/jemr.12.6.9, PMID: 33828757 PMC7962684

[ref48] Kang S. L. Shaikh A. G. Ghasia F. F. (2018). Vergence and strabismus in neurodegenerative disorders. Front. Neurol. 9:299. doi: 10.3389/fneur.2018.00299, PMID: 29867716 PMC5964131

[ref49] Kapoula Z. Bucci M. P. Eggert T. Garraud L. (1997). Impairment of the binocular coordination of saccades in strabismus. Vis. Res. 37, 2757–2766. doi: 10.1016/S0042-6989(97)00064-3, PMID: 9373674

[ref50] King W. M. (2011). Binocular coordination of eye movements--Hering’s law of equal innervation or uniocular control? Eur. J. Neurosci. 33, 2139–2146. doi: 10.1111/j.1460-9568.2011.07695.x, PMID: 21645107 PMC3111934

[ref51] Krauzlis R. J. (2003). Neuronal activity in the rostral superior colliculus related to the initiation of pursuit and saccadic eye movements. J. Neurosci. 23, 4333–4344. doi: 10.1523/JNEUROSCI.23-10-04333.2003, PMID: 12764122 PMC6741111

[ref52] Lee Y. Y. Granger-Donetti B. Chang C. Alvarez T. L. (2009). Sustained convergence induced changes in phoria and divergence dynamics. Vis. Res. 49, 2960–2972. doi: 10.1016/j.visres.2009.09.013 19781567

[ref53] Leigh R. J. Riley D. E. (2000). Eye movements in parkinsonism: it’s saccadic speed that counts. Neurology 54, 1018–1019. doi: 10.1212/WNL.54.5.1018, PMID: 10720267

[ref54] Lepore F. E. (2006). Parkinson’s disease and diplopia. Neuro-Ophthalmology 30, 37–40. doi: 10.1080/01658100600742838

[ref55] Matsumura M. Kojima J. Gardiner T. W. Hikosaka O. (1992). Visual and oculomotor functions of monkey subthalamic nucleus. J. Neurophysiol. 67, 1615–1632. doi: 10.1152/jn.1992.67.6.1615, PMID: 1629767

[ref56] May P. J. Porter J. D. Gamlin P. D. (1992). Interconnections between the primate cerebellum and midbrain near-response regions. J. Comp. Neurol. 315, 98–116. doi: 10.1002/cne.903150108, PMID: 1371782

[ref57] Mays L. E. (1984). Neural control of vergence eye movements: convergence and divergence neurons in midbrain. J. Neurophysiol. 51, 1091–1108. doi: 10.1152/jn.1984.51.5.1091, PMID: 6726313

[ref58] Mays L. E. Zhang Y. Thorstad M. H. Gamlin P. D. (1991). Trochlear unit activity during ocular convergence. J. Neurophysiol. 65, 1484–1491. doi: 10.1152/jn.1991.65.6.1484, PMID: 1875256

[ref59] Monakow K. H. V. Akert K. Knzle H. (1978). Projections of the precentral motor cortex and other cortical areas of the frontal lobe to the subthalamic nucleus in the monkey. Exp. Brain Res. 33, 395–403. doi: 10.1007/BF00235561, PMID: 83239

[ref60] Naumann W. Gogarten J. Schönfeld S. Klostermann F. Marzinzik F. Schindlbeck K. A. (2021). Diplopia in Parkinson’s disease: indication of a cortical phenotype with cognitive dysfunction? Acta Neurol. Scand. 144, 440–449. doi: 10.1111/ane.13479, PMID: 34096617

[ref61] Ooi T. L. He Z. J. (2015). Space perception of strabismic observers in the real world environment. Invest. Ophthalmol. Vis. Sci. 56, 1761–1768. doi: 10.1167/iovs.14-15741, PMID: 25698702 PMC4358738

[ref62] Otero-Millan J. Optican L. M. Macknik S. L. Martinez-Conde S. (2018). Modeling the triggering of saccades, microsaccades, and saccadic intrusions. Front. Neurol. 9:346. doi: 10.3389/fneur.2018.00346, PMID: 29892256 PMC5985689

[ref63] Pallus A. C. Walton M. M. G. Mustari M. J. (2018). Response of supraoculomotor area neurons during combined saccade-vergence movements. J. Neurophysiol. 119, 585–596. doi: 10.1152/jn.00193.2017 29142092 PMC5867375

[ref64] Qing Y. Kapoula Z. (2004). Saccade-vergence dynamics and interaction in children and in adults. Exp. Brain Res. 156, 212–223. doi: 10.1007/s00221-003-1773-1, PMID: 15344851

[ref65] Racette B. A. Gokden M. Tychsen L. Perlmutter J. S. (1999). Convergence insufficiency in idiopathic Parkinson’s disease responsive to levodopa. Strabismus 7, 169–174. doi: 10.1076/stra.7.3.169.636 10520242

[ref66] Ramat S. Leigh R. J. Zee D. S. Optican L. M. (2005). Ocular oscillations generated by coupling of brainstem excitatory and inhibitory saccadic burst neurons. Exp. Brain Res. 160, 89–106. doi: 10.1007/s00221-004-1989-8, PMID: 15289966

[ref67] Ramat S. Leigh R. J. Zee D. S. Optican L. M. (2006). What clinical disorders tell us about the neural control of saccadic eye movements. Brain 130, 10–35. doi: 10.1093/brain/awl309, PMID: 17121745

[ref68] Repka M. X. Claro M. C. Loupe D. N. Reich S. G. (1996). Ocular motility in Parkinson’s disease. J. Pediatr. Ophthalmol. Strabismus 33, 144–147. doi: 10.3928/0191-3913-19960501-04 8771514

[ref69] Santos-García D. Naya Ríos L. de Deus Fonticoba T. Cores Bartolomé C. García Roca L. Feal Painceiras M.. (2021). Diplopia is frequent and associated with motor and non-motor severity in Parkinson’s disease: results from the COPPADIS cohort at 2-year follow-up. Diagnostics (Basel) 11:2380. doi: 10.3390/diagnostics11122380 34943619 PMC8700703

[ref70] Santos-García D. Aneiros-Díaz A. Macías-Arribi M. Llaneza M. Abella-Corral J. (2010). Sensory symptoms in Parkinson’s disease. Rev. Neurol. 50, S65–S74. doi: 10.33588/rn.50S02.2009742 20205145

[ref71] Sauerbier A. Ray Chaudhuri K. (2013). Parkinson’s disease and vision. Basal Ganglia 3, 159–163. doi: 10.1016/j.baga.2013.05.002

[ref72] Schindlbeck K. A. Schönfeld S. Naumann W. Friedrich D. J. Maier A. Rewitzer C.. (2017). Characterization of diplopia in non-demented patients with Parkinson’s disease. Parkinsonism Relat. Disord. 45, 1–6. doi: 10.1016/j.parkreldis.2017.09.024, PMID: 28993094

[ref73] Schroeder T. L. Rainey B. B. Goss D. A. Grosvenor T. P. (1996). Reliability of and comparisons among methods of measuring dissociated phoria. Optom. Vis. Sci. 73, 389–397. doi: 10.1097/00006324-199606000-00006, PMID: 8807650

[ref74] Searle A. Rowe F. J. (2016). Vergence neural pathways: a systematic narrative literature review. Neuroophthalmology 40, 209–218. doi: 10.1080/01658107.2016.1217028, PMID: 27928407 PMC5122972

[ref75] Semmlow J. L. Yaramothu C. Alvarez T. L. (2019). Dynamics of the disparity vergence fusion sustain component. J. Eye Mov. Res. 12:10.16910/jemr.12.4.11. doi: 10.16910/jemr.12.4.11 PMC7173722 32318253

[ref76] Semmlow J. Yaramothu C. Scheiman M. Alvarez T. (2021). Vergence fusion sustaining oscillations. J. Eye Mov. Res. 14:10.16910/jemr.14.1.4. doi: 10.16910/jemr.14.1.4, PMID: 34221249 PMC8247062

[ref77] Shaikh A. G. Ghasia F. F. (2017). Fixational saccades are more disconjugate in adults than in children. PLoS One 12:e0175295. doi: 10.1371/journal.pone.0175295, PMID: 28406944 PMC5391133

[ref78] Shaikh A. G. Ghasia F. F. (2019). Saccades in Parkinson’s disease: hypometric, slow, and maladaptive. Prog. Brain Res. 249, 81–94. doi: 10.1016/bs.pbr.2019.05.001, PMID: 31325999

[ref79] Shaikh A. G. Meng H. Angelaki D. E. (2004). Multiple reference frames for motion in the primate cerebellum. J. Neurosci. 24, 4491–4497. doi: 10.1523/JNEUROSCI.0109-04.2004, PMID: 15140919 PMC6729386

[ref80] Shook B. L. Schlag-Rey M. Schlag J. (1990). Primate supplementary eye field: I. Comparative aspects of mesencephalic and pontine connections. J. Comp. Neurol. 301, 618–642. doi: 10.1002/cne.903010410, PMID: 2273101

[ref81] Sieger T. Bonnet C. Serranová T. Wild J. Novák D. Růžička F.. (2013). Basal ganglia neuronal activity during scanning eye movements in Parkinson’s disease. PLoS One 8:e78581. doi: 10.1371/journal.pone.0078581, PMID: 24223158 PMC3819366

[ref82] Smilowska K. Wowra B. Slawek J. (2020). Double vision in Parkinson’s disease: a systematic review. Neurol. Neurochir. Pol. 54, 502–507. doi: 10.5603/PJNNS.a2020.0092 33300115

[ref83] Stanton G. B. Goldberg M. E. Bruce C. J. (1988). Frontal eye field efferents in the macaque monkey: II. Topography of terminal fields in midbrain and pons. J. Comp. Neurol. 271, 493–506. doi: 10.1002/cne.902710403, PMID: 2454971

[ref84] Steinman R. M. Cushman W. B. Martins A. J. (1982). The precision of gaze. A review. Hum. Neurobiol. 1, 97–109. PMID: 6764462

[ref85] Subramanian V. Jost R. M. Birch E. E. (2013). A quantitative study of fixation stability in amblyopia. Invest. Ophthalmol. Vis. Sci. 54, 1998–2003. doi: 10.1167/iovs.12-11054, PMID: 23372053 PMC3604910

[ref86] Upadhyaya S. Das V. E. (2019). Response properties of cells within the rostral superior colliculus of strabismic monkeys. Invest. Ophthalmol. Vis. Sci. 60, 4292–4302. doi: 10.1167/iovs.19-27786, PMID: 31618766 PMC6996666

[ref87] Upadhyaya S. Meng H. Das V. E. (2017). Electrical stimulation of superior colliculus affects strabismus angle in monkey models for strabismus. J. Neurophysiol. 117, 1281–1292. doi: 10.1152/jn.00437.2016, PMID: 28031397 PMC5349331

[ref88] Urwyler P. Nef T. Killen A. Collerton D. Thomas A. Burn D.. (2014). Visual complaints and visual hallucinations in Parkinson’s disease. Parkinsonism Relat. Disord. 20, 318–322. doi: 10.1016/j.parkreldis.2013.12.009, PMID: 24405755

[ref89] van der Lijn I. de Haan G. A. van der Feen F. E. Huizinga F. Fuermaier A. B. M. van Laar T.. (2022). Correction: the screening visual complaints questionnaire (SVCq) in people with Parkinson’s disease-confirmatory factor analysis and advice for its use in clinical practice. PLoS One 17:e0278279. doi: 10.1371/journal.pone.0278279, PMID: 36413555 PMC9681063

[ref90] Van Horn M. R. Waitzman D. M. Cullen K. E. (2013). Vergence neurons identified in the rostral superior colliculus code smooth eye movements in 3D space. J. Neurosci. 33, 7274–7284. doi: 10.1523/JNEUROSCI.2268-12.2013, PMID: 23616536 PMC6619582

[ref91] van Leeuwen A. F. Collewijn H. Erkelens C. J. (1998). Dynamics of horizontal vergence movements: interaction with horizontal and vertical saccades and relation with monocular preferences. Vis. Res. 38, 3943–3954. doi: 10.1016/S0042-6989(98)00092-3, PMID: 10211386

[ref92] Visser F. Vlaar A. M. M. Borm C. D. J. M. Apostolov V. Lee Y. X. Notting I. C.. (2019). Diplopia in Parkinson’s disease: visual illusion or oculomotor impairment? J. Neurol. 266, 2457–2464. doi: 10.1007/s00415-019-09430-w 31214767

[ref93] Walton M. M. G. Pallus A. Fleuriet J. Mustari M. J. Tarczy-Hornoch K. (2017). Neural mechanisms of oculomotor abnormalities in the infantile strabismus syndrome. J. Neurophysiol. 118, 280–299. doi: 10.1152/jn.00934.2016, PMID: 28404829 PMC5498729

[ref94] Wu C. C. Cao B. Dali V. Gagliardi C. Barthelemy O. J. Salazar R. D.. (2018). Eye movement control during visual pursuit in Parkinson’s disease. PeerJ 6:e5442. doi: 10.7717/peerj.5442, PMID: 30155357 PMC6109371

[ref95] Zee D. S. Fitzgibbon E. J. Optican L. M. (1992). Saccade-vergence interactions in humans. J. Neurophysiol. 68, 1624–1641. doi: 10.1152/jn.1992.68.5.1624, PMID: 1479435

[ref96] Zee D. S. Levi L. (1989). Neurological aspects of vergence eye movements. Rev. Neurol. (Paris) 145, 613–620. 2682935

[ref97] Zhang Y. Mays L. E. Gamlin P. D. (1992). Characteristics of near response cells projecting to the oculomotor nucleus. J. Neurophysiol. 67, 944–960. doi: 10.1152/jn.1992.67.4.944, PMID: 1588393

